# Ginsenoside Rh1 Inhibits Angiotensin II-Induced Vascular Smooth Muscle Cell Migration and Proliferation through Suppression of the ROS-Mediated ERK1/2/p90RSK/KLF4 Signaling Pathway

**DOI:** 10.3390/antiox11040643

**Published:** 2022-03-27

**Authors:** Diem Thi Ngoc Huynh, Yujin Jin, Dung Van Nguyen, Chang-Seon Myung, Kyung-Sun Heo

**Affiliations:** 1 College of Pharmacy and Institute of Drug Research and Development, Chungnam National University, 99 Daehak-ro, Yuseong-Gu, Daejeon 34134, Korea; diem87@o.cnu.ac.kr (D.T.N.H.); 201850535@o.cnu.ac.kr (Y.J.); nvdung.pharmacy98@o.cnu.ac.kr (D.V.N.); cm8r@cnu.ac.kr (C.-S.M.); 2 Department of Pharmacy, Da Nang University of Medical Technology and Pharmacy, Da Nang 550000, Vietnam

**Keywords:** angiotensin II, ginsenoside Rh1, KLF4, p90RSK, reactive oxygen species, smooth muscle cell

## Abstract

Vascular smooth muscle cell (VSMC) proliferation and migration play key roles in the progression of atherosclerosis and restenosis. A variety of ginsenosides exert various cardiovascular benefits. However, whether and how ginsenoside Rh1 (Rh1) inhibits VSMC dysfunction remain unclear. Here, we investigated the inhibitory effects of Rh1 on rat aortic smooth muscle cell (RASMC) migration and proliferation induced by angiotensin II (Ang II) and the underlying mechanisms. Cell proliferation and migration were evaluated using sulforhodamine B and wound-healing assay. The molecular mechanisms were investigated using Western blotting, quantitative reverse-transcription polymerase chain reaction analysis, immunofluorescence staining, and luciferase assay. Reactive oxygen species (ROS) production was measured using dihydroethidium and MitoSOX staining. We found that Rh1 dose-dependently suppressed Ang II-induced cell proliferation and migration. Concomitantly, Ang II increased protein levels of osteopontin, vimentin, MMP2, MMP9, PCNA, and cyclin D1, while these were reduced by Rh1 pretreatment. Notably, Ang II enhanced both the protein expression and promoter activity of KLF4, a key regulator of phenotypic switching, whereas pretreatment with Rh1 reversed these effects. Mechanistically, the effects of Rh1 on VSMC proliferation and migration were found to be associated with inhibition of ERK1/2/p90RSK signaling. Furthermore, the inhibitory effects of Rh1 were accompanied by inhibition of ROS production. In conclusion, Rh1 inhibited the Ang II-induced migration and proliferation of RASMCs by suppressing the ROS-mediated ERK1/2/p90RSK signaling pathway.

## 1. Introduction

Vascular smooth muscle cells (VSMCs) are the main components of the media layer of arteries [1]. The aberrant transformations in their complex structure and functions contribute to the development of various vascular diseases, such as atherosclerosis, restenosis, hypertension, aneurysms, and vascular aging [2]. In particular, the phenotypic switching, a shift from a contractile phenotype to a synthetic state, in VSMCs is stimulated by a variety of molecules and factors, such as growth factors, inflammatory stimuli, and oxidative stress, through diverse signaling pathways, including rat sarcoma virus (Ras)/rapidly accelerated fibrosarcoma (Raf)/mitogen-activated protein kinase/ERK kinase (MEK)/extracellular signal-regulated kinase (ERK), phosphatidylinositol 3-kinase (PI3K)/protein kinase B (Akt), nuclear factor kappa B, and Janus kinase (JAK)2/signal transducer and activator of transcription (STAT)3 pathways [3]. This results in increases of cell proliferation and migration, which have been determined to be highly linked to the pathogenesis of vascular diseases [2,4]. Therefore, targeting VSMC phenotypic change, as well as the proliferation and migration of these cells, is a notable approach to the treatment of various vascular diseases.

Angiotensin II (Ang II), a peptide hormone of the renin-angiotensin-aldosterone system, is considered a risk factor for the cardiovascular system [5]. Ang II receptors have two main isoforms: Ang II type 1 receptor (AT1R) and Ang II type 2 receptor (AT2R). It has been reported that the majority of Ang II effects in the adult tissues result from interactions of the AT1R [6]. In addition, emerging data have reported that Ang II activates various signaling pathways, such as the MAPK and PI3K signaling pathways, through the AT1R, to induce VSMC proliferation and migration [7,8]. Furthermore, this hormone promotes VSMC proliferation and migration through the production of reactive oxygen species (ROS) [9]. Our previous study also demonstrated that p90 ribosomal S6 kinase (p90RSK), a downstream effector of ERK1/2, plays an important role in the Ang II-stimulated proliferation and migration of VSMCs [9]. In addition, a recent study revealed that Ang II increased expression of KLF4 through the ROS-mediated ERK1/2 signaling pathway [10].

Emerging data have revealed that oxidative stress is associated with the pathogenic mechanisms and the development of cardiovascular diseases [11]. NADPH oxidases (NOXs), which are widely expressed in VSMCs, are stimulated by various factors, such as Ang II and hypoxia [11]. The activation of NOXs leads to ROS generation, including superoxide anions, hydroxyl radicals, and hydrogen peroxide [11]. The stimulation of VSMC proliferation and migration by ROS has been widely reported [11]. Interestingly, superoxide anions, but not hydrogen peroxide, rapidly activate PKC and ERK1/2 signaling in VSMCs [12].

The phenotypic switching of VSMCs has been reported to be associated with cell proliferation and migration. Notably, KLF4 plays a key role in regulating VSMC phenotypic switching [1]. In fact, previous studies revealed that, under stimulation of various agents, including platelet-derived growth factor (PDGF) and oxidized phospholipids, the protein expression and transcriptional activity of KLF4 were increased, which led to repression of VSMC contractile marker genes [13]. In addition, an increase in the matrix metalloproteinase (MMP) activity is associated with the degradation of the extracellular matrix, which leads to VSMC migration [14]. Indeed, a number of studies have demonstrated that MMP2 and MMP9 play critical roles in VSMC migration [15]. 

Exploring new therapeutic agents from natural sources is an important strategy in drug discovery. Ginsenosides, the major bioactive components of ginseng, have been reported to exert a broad range of biological activities, such as anti-cancer, anti-aging, anti-inflammatory, anti-diabetic, neuroprotective, and cardiovascular-protective activities [16,17,18,19,20,21,22,23]. In particular, ginsenoside Rh1 (Rh1) has been found to possess potential benefits against several cardiovascular dysfunctions, such as myocardial injury and vascular leakage [24,25]. Notably, a variety of ginsenosides have been shown to exert inhibitory effects on VSMC proliferation and migration via different mechanisms. In particular, ginsenoside Rb3 suppresses the proliferation of VSMCs in response to Ang II by blocking the transition of the cell cycle progression from G0/G1 to the S phase [26]. In addition, the inhibitory effect of ginsenoside Rg1 on VSMC proliferation has been demonstrated to be related to the inhibition of the tumor necrosis factor-α-activated PKCζ pathway and the increase of NO formation in response to PDGF-BB stimulation [27,28]. Particularly, ginsenoside Rg3 stereoisomers inhibit both the proliferation and migration of VSMCs in diabetic atherosclerosis [29]. Additionally, compound K, an intestinal metabolite of ginsenosides, decreases PDGF-BB-stimulated VSMC proliferation and migration through G1 arrest [30]. However, the effects of Rh1 on VSMC dysfunction remain unclear. This study aimed to discover the effects of Rh1 on Ang II-induced VSMC migration and proliferation and to elucidate the mechanisms underlying these effects.

## 2. Materials and Methods

### 2.1. Antibodies and Reagents

Rabbit anti-phospho-ERK1/2 (#4370), rabbit anti-ERK1/2 (#4695), rabbit anti-cyclin D1 (#2978), rabbit anti-phospho-p90RSK (#12032S), rabbit anti-RSK1 (#8408S), rabbit anti-phospho-Akt ser473 (#9271), rabbit anti-Akt (#9272), rabbit anti-vimentin (#5741), and rabbit α-SMA (#19245) antibodies were purchased from Cell Signaling Technology, Inc. (Danvers, MA, USA). Rabbit anti-MMP2 (#AB9015) and rabbit anti-MMP9 (#AB19016) antibodies were purchased from Merck Millipore (Burlington, MA, USA). Mouse anti-PCNA (#sc-56), mouse anti-OPN (#sc-73631), and mouse anti-KLF4 (#sc-393462) antibodies were purchased from Santa Cruz Biotechnology Inc. (Dallas, TX, USA). Rabbit anti-GAPDH (#LF-PA0018) was purchased from AbFrontier (Seoul, Korea). Mouse anti-α-tubulin antibody, crystal violet (#C0775), 2′,7′-dichlorodihydrofluorescein diacetate (DCF-DA), sulforhodamine B sodium salt (SRB; #S1402), trichloroacetic acid (TCA; #T6399), Mito-TEMPO (MT; #SML0737), and Rh1 were purchased from Sigma-Aldrich (St. Louis, MO, USA). Goat anti-rabbit IgG second antibody (#31460) and goat anti-mouse IgG second antibody (#31430) were obtained from Invitrogen (Carlsbad, CA, USA). Phosphate-buffered saline (PBS; #EBA-1105) was purchased from Elpisbio (Daejeon, South Korea). Diphenyleneiodonium chloride (DPI; #0504) and apocynin (APO; #4663) were obtained from Tocris Bio-science (Ellisville, MO, USA). 

### 2.2. Cell Culture 

Rat aortic smooth muscle cells (RASMCs) were obtained from the American Type Culture Collection (Manassas, VA, USA). The cells were grown in the conditions described in our previous report [9]. The characteristics and contractile phenotype of VSMCs are maintained up to passage 15, which were confirmed by immunofluorescence staining for alpha-smooth muscle actin (α-SMA) (Figure S1). For experiments, the cells were used at passages 5–10. 

### 2.3. Wound-Healing Assay 

Wound-healing assay was utilized to analyze cell migration, according to the previous report [4]. Briefly, when the cells reached approximately 90% confluence, wound areas were created with a p200 pipet tip. After the indicated treatment, 4% formalin was used to fix the cells, and then 0.5% crystal violet was used to stain the cells. Finally, an Olympus DP72 digital microscope (Olympus cooperation, Tokyo, Japan) was utilized to capture images. 

### 2.4. Sulforhodamine B (SRB) Assay

SRB assay was implemented to examine cell viability, as described previously [17]. Briefly, RASMCs were seeded in 96-well plates. At the end of the indicated treatment, 10% TCA was used to fix the cells. Next, after being washed, the cells were air-dried at room temperature (RT). Then, 0.057% SRB was applied to stain the cells for 30 min. After washing with 1% acetic acid and drying, 10 mM Tris base solution (pH 10.5) was used to dissolve the dye, and a microplate reader (Tecan, Männedorf, Switzerland) was operated at 510 nm to measure absorbance.

### 2.5. Western Blot Analysis

Western blot was carried out to analyze protein expression according to the previous description [21]. Briefly, at the end of treatment, cells were washed and lysed with a 2× sodium dodecyl sulfate (SDS) buffer. Cell lysates were resolved by sodium dodecyl sulfate polyacrylamide gel electrophoresis (SDS-PAGE) and transferred onto a nitrocellulose membrane. After being blocked with 5% skimmed milk for 1 h at room temperature (RT), the membranes were incubated with primary antibodies (1:1000 dilution) at 4 °C overnight, and then incubated with secondary antibodies (1:2500 dilution) for 90 min at RT. Finally, proteins on membranes were visualized using the enhanced chemiluminescence detection reagents. Original Western Blot figures can be found from File S1.

### 2.6. Real-Time Quantitative Reverse Transcription-Polymerase Chain Reaction (qRT-PCR)

Tri-RNA reagent (Favorgen, Pingtung, China) was used to extract total RNA from the cells. Total RNA (1 µg) was used for cDNA synthesis using reverse transcription 5X master mix. Specific primers and iQ™ SYBR green supermix (Bio-Rad Inc., Hercules, CA, USA) were used for the quantification of mRNA expression through a CFX Connect™ (Bio-Rad Inc.). Finally, the 2^−∆∆Ct^ method was used to evaluate the mRNA levels of individual genes by normalizing to β-actin. The following primers were used: MMP2 (rat), forward-5′-GATACCCCAAGCCACTG-3′ and reverse-5′-TCCAAACTTCACGCTCTT-3′; MMP9 (rat), forward-5′-CAGACCAAGGGTACAGCCTGTT-3′ and reverse-5′-AGCGCATGGCCGAACTC-3′; α-SMA (rat), forward-5′-CATCACCAACTGGGACGACA-3′ and reverse-5′-TCCGTTAGCAAGGTCGGATG-3′; OPN (rat), forward-5′-GCT CTCAAGGTCATCCCAGTTG-3′ and reverse-5′-TGTTTCCACGCTTGGTTCACT-3′; vimentin (rat), forward-5′-AGGGGAGGAGAGCAGGATTT-3′ and reverse-5′-GGAGTGGGTGTCAACCAGAG-3′; β-actin (rat), forward-5′-ATGGATGACGATATCGCTGCG-3′ and reverse-5′-CAGGGTCAGGATGCCTCTCTT-3′.

### 2.7. Dihydroethidium (DHE) Staining

The treated cells were stained with 10 µM DHE (#D11347, Invitrogen) at 37 °C for 30 min. After washing with PBS, a fluorescence microscope (Olympus IX71, Tokyo, Japan) was operated to monitor fluorescence. 

### 2.8. MitoSOX Staining

The treated cells were incubated with MitoSOX Red (4 µM) at 37 °C for 10 min. Next, after washing with PBS, 4% formaldehyde was used to fix the cells for 10 min. Finally, DAPI mounting solution was applied to counterstain nuclei, and images were taken by a laser scanning confocal spectral microscope (K1-Fluo, Nanoscope systems, Daejeon, Korea). 

### 2.9. Immunofluorescence Assay

Following fixation with 4% formaldehyde, 0.2% Triton X-100 was used to permeate the cells. After being blocked by 3% BSA, cells were incubated with primary antibody (4 °C, overnight) with a dilution of rabbit anti-α-SMA antibody (#19245, Cell Signaling Technology Inc., Danvers, MA, USA) at 1:800 and dilution of mouse anti-PCNA antibody (#sc-56, Santa Cruz Biotechnology Inc., Dallas, TX, USA) at 1:50. Then, the cells were further incubated with fluorescent secondary antibodies, including goat anti-rabbit IgG, highly cross-adsorbed secondary antibody Alexa Fluor 488 (#A11034, Invitrogen), and goat anti-mouse IgG highly cross-adsorbed secondary antibody Alexa Fluor 568 (#A11031, Invitrogen) with 1:600 dilutions, at RT for 1 h. Subsequently, a laser scanning confocal spectral microscope was utilized to capture fluorescent images.

### 2.10. Luciferase Reporter Gene Assay

Promoter activity was analyzed according to the previous description [9]. Briefly, cells were transfected with pKLF4-Luc and pRL-CMV renilla plasmid (normalization control) using Solfect transfection reagent (BSX-SFA-001, Biosolyx, Daegu, Korea). At the end of treatment, the cells were lysed with passive lysis buffer, and reporter activity was measured with a dual-luciferase kit (E1960, Promega, Madison, WI, USA) by a Glomax luminometer (Promega BioSystems Sunnyvale Inc., Sunnyvale, CA, USA). 

### 2.11. Statistical Analysis

GraphPad Prism 5 (GraphPad Software Inc., San Diego, CA, USA) was used for statistical analysis. Data were analyzed by one-way analysis of variance (ANOVA), followed by a Turkey’s multiple comparison test or Bonferroni’s multiple comparison test. Additionally, a Kruskal–Wallis test, followed by Dunn’s multiple comparison test, was also used to evaluate the results. At least three independent replications were carried out. Difference was considered significant when *p* value was <0.05. All results were represented as the mean ± standard error of the mean (SEM). 

## 3. Results

### 3.1. Rh1 Inhibits Ang II-Induced RASMC Migration 

**Figure 1:**
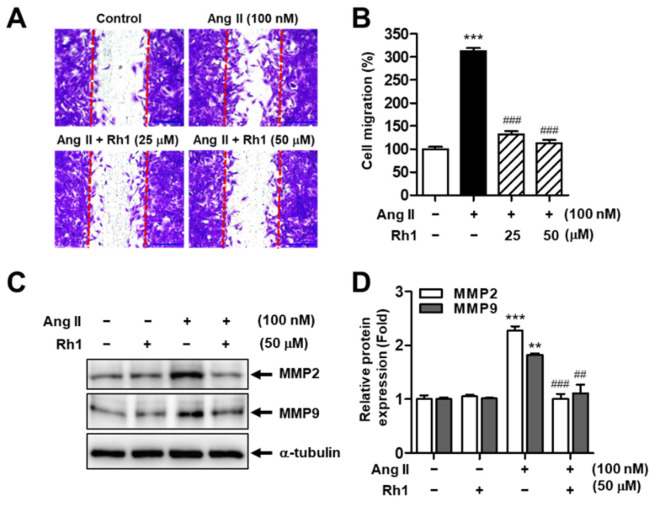
Rh1 inhibits VSMC migration promoted by Angiotensin II (Ang II). Cells were pretreated with Rh1 for 3 h, before treatment with 100 nM Ang II for 24 h. (**A**) Wound-healing assay indicates cell migration. The red lines indicate a clear zone after scratching (0 h). (**B**) The quantification of cell migration was carried out by Image J software. (**C**,**D**) Western blot analysis was used to analyze the expression levels of the indicated proteins in the cells. (**B**,**D**) Results are represented as means ± SEM (*n* = 5), ** *p* < 0.01 and *** *p* < 0.001 compared with the control, ## *p* < 0.01 and ### *p* < 0.001 compared with the Ang II-treated sample. GAPDH: glyceraldehyde 3-phosphate dehydrogenase, MMP: matrix metalloproteinase.

To explore whether Rh1 exerts inhibitory effects on Ang II-induced cell migration, RASMCs were preincubated with Rh1 (25 and 50 µM) for 3 h before exposure to Ang II (100 nM) for 24 h. We found that Ang II dramatically promoted cell migration, which increased to 312 ± 6.57% compared with the control samples (*p* < 0.001). However, pretreatment with Rh1 dose-dependently rescued these effects; in particular, at a concentration of 50 µM, Rh1 almost suppressed the effect of Ang II on cell migration, reaching 113.2 ± 6.67% (*p* < 0.001) (Figure 1A,B). Consistently, migration-related molecules, such as MMP2 and MMP9, were augmented in response to Ang II stimulation. However, pretreatment with Rh1 completely reversed these effects (Figure 1C,D). 

### 3.2. Rh1 Inhibits Ang II-Induced RASMC Proliferation

**Figure 2:**
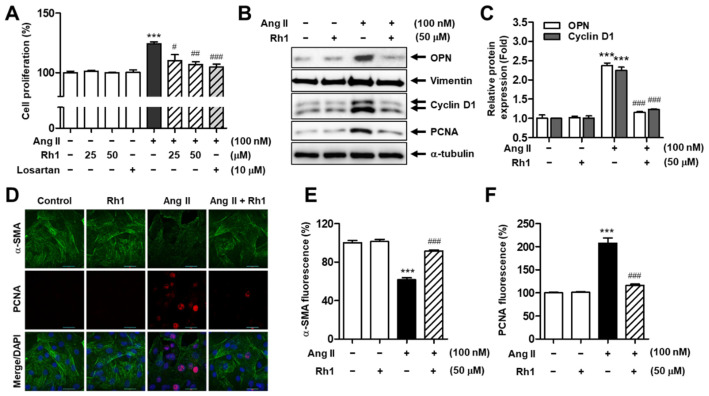
Rh1 inhibits the cell proliferation induced by Ang II. (**A**) Cell proliferation was evaluated by the sulforhodamine B (SRB) assay. (**B**,**C**) Western blot analysis was used to analyze indicated protein levels in the whole-cell lysates. (**D**–**F**) The immunofluorescence analysis was performed after immunostaining using indicated antibodies. DAPI co-staining was used to indicate the nuclei of the cells. Representative photographs are shown at a magnification of ×200 (scale bar: 30 μm) (**D**). The relative fluorescence intensity of α-SMA (**E**) and that of PCNA (**F**) were quantified by Image J software. (**A**,**C**,**E**,**F**) Data are expressed as the mean ± SEM (*n* = 4), *** *p* < 0.001 compared with the control, # *p* < 0.05, ## *p* < 0.01 and ### *p* < 0.001 compared with the Ang II-treated sample.

We next investigated the effect of Rh1 on Ang II-induced cell proliferation using SRB assay. The increase in cell proliferation stimulated by Ang II (% of increase vs. control group: 24.3 ± 1.47%, *p* < 0.001) was gradually inhibited by the pretreatment with Rh1, while the incubation with only Rh1 (25 and 50 mM) did not affect the cell viability (Figure 2A). Particularly, the decrease in cell proliferation by 50 µM Rh1 (% of decrease vs. Ang II group: 17.4 ± 2.38%, *p* < 0.01) was relatively similar to that of 10 µM losartan (% of decrease vs. Ang II group: 19.6 ± 2.42%, *p* < 0.001). Consistently, Ang II increased the protein levels of cyclin D1 and proliferating cell nuclear antigen (PCNA), proliferation markers. However, the pretreatment with Rh1 reversed these effects (Figure 2B–D).

The decrease in contractile marker expressions and the increase in synthetic marker levels and proliferation rate are the main features of VSMC phenotypic switching [31]. As indicated in Figure 2B,C, in addition to the elevated protein expression of cyclin D1 and PCNA, Ang II significantly increased protein expression of synthetic phenotype-related molecules, including osteopontin (OPN) and vimentin, whereas Rh1 pretreatment nearly rescued these effects. In addition, the effect of Rh1 on Ang II-induced shift towards the synthetic phenotype was observed through the immunofluorescence staining of a contractile marker, α-SMA, and a proliferation marker, PCNA. Consistently, Ang II treatment led to a significant reduction in α-SMA expression to 61.69 ± 1.90% (*p* < 0.001) and a remarkable increase in PCNA expression to 207.5 ± 11.12% (*p* < 0.001). Meanwhile, Rh1 pretreatment completely reversed these detrimental impacts of Ang II on RASMCs (Figure 2D–F). These results suggest that Rh1 exerts a potential effect in preventing Ang II-induced phenotypic switching and proliferation of VSMCs.

### 3.3. Rh1 Inhibits Ang II-Activated ERK1/2/p90RSK Signaling Pathway in RASMCs

**Figure 3:**
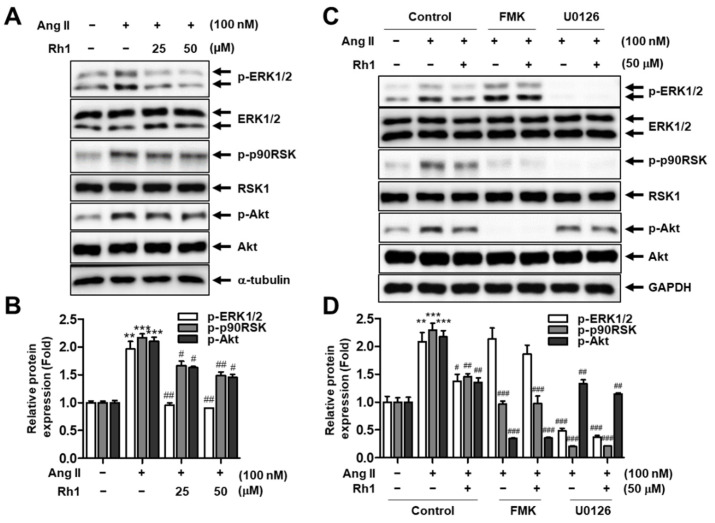
Rh1 inhibits the activation of the ERK1/2/p90RSK signaling pathway induced by Ang II. (**A**,**B**) RASMCs were pretreated with Rh1 (25 and 50 µM) for 1 h before being incubated with Ang II (100 nM) for 1 h. (**C**,**D**) The cells were preincubated with 20 µM FMK or 10 µM U0126 and/or 50 µM Rh1 for 1 h, and then exposed to Ang II for 1 h. (**A**–**D**) Western blot analysis was used to analyze the levels of the indicated proteins in the whole-cell lysates. (**B**,**D**) Results are represented as means ± SEM (*n* = 4), ** *p* < 0.01, and *** *p* < 0.001 compared with the control, # *p* < 0.05, ## *p* < 0.01, and ### *p* < 0.001 compared with the Ang II-treated sample. FMK: p90RSK inhibitor, U0126: ERK1/2 inhibitor.

The MAPK signaling pathway is closely associated with VSMC migration and proliferation [9]. Therefore, to clarify the signaling pathway responsible for the inhibitory effect of Rh1 on Ang II-stimulated VSMC proliferation and migration, the MAPK signaling pathway was investigated. The results revealed that the Rh1 pretreatment significantly inhibited the Ang II-induced ERK1/2, p90RSK, and Akt phosphorylation (Figure 3A,B). The inhibition of ERK1/2 activation by U0126 completely blocked the Ang II-induced phosphorylation of p90RSK, but FMK, a specific inhibitor of p90RSK, did not change ERK1/2 activation in Ang II-stimulated RASMCs (Figure 3C,D). This suggests that p90RSK is downstream of ERK1/2 in Ang II-stimulated VSMCs, and that Rh1 treatment downregulated this signaling axis. 

### 3.4. Rh1 Inhibits Ang II-Induced Cell Proliferation and Migration by Regulating KLF4 in VSMCs

**Figure 4:**
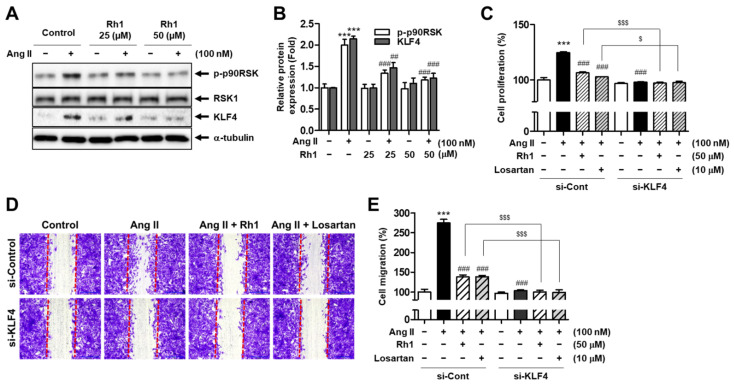
Rh1 inhibits the Ang II-induced proliferation and migration of VSMCs by regulating KLF4. (**A**,**B**) RASMCs were pretreated with Rh1 (25 and 50 µM) before 24 h of exposure to Ang II (100 nM). Western blot analysis was used to analyze the levels of indicated proteins in the whole-cell lysates. (**C**–**E**) The cells were transfected with 100 nM KLF4 siRNA (si-KLF4) or control siRNA (si-Cont) and maintained for 24 h. The cells were then pretreated with Rh1 (50 µM) or losartan (10 µM) for 3 h, which was followed by treatment with 100 nM Ang II for 24 h. Cell proliferation was examined by the SRB assay (**C**), and cell migration was evaluated by the wound-healing assay (**D**,**E**). (**B**,**C**,**E**) The data are presented as the mean ± SEM (*n* = 3), *** *p* < 0.001 compared with the control. ## *p* < 0.01 and ### *p* < 0.001 compared with the Ang II-treated sample. $ *p* < 0.05, and $$$ *p* < 0.001 compared with the corresponding sample.

KLF4 is a key regulator of SMC phenotypic switching [32]. Because we found that Rh1 had an effect on Ang II-induced changes in the protein levels of contractile and synthetic markers, the protein expression of KLF4 was examined. RASMCs exhibited a significant increase in the protein expression of KLF4 to 214.3 ± 4.53% (*p* < 0.001) in response to Ang II stimulation, and this effect was decreased by the Rh1 pretreatment to 122.4 ± 8.04% (*p* < 0.001) (Figure 4A,B). Next, to clarify the role of KLF4 regulation in the effect of Rh1 on Ang II-induced VSMC proliferation and migration, KLF4 siRNA was used to induce knockdown of KLF4, and cell migration and proliferation were then validated. The results showed that KLF4 depletion completely inhibited cell migration and proliferation promoted by Ang II, indicating that KLF4 plays a key role in regulating cell migration and proliferation induced by Ang II in VSMCs (Figure 4C–E). Furthermore, the knockdown of KLF4 further enhanced the inhibitory effect of Rh1 on cell proliferation and migration (Figure 4C–E). In addition, the inhibitory effect of 50 µM Rh1 was similar to that of 10 µM losartan. These data indicate that Rh1 inhibits the proliferation and migration of Ang II-stimulated VSMCs through the regulation of KLF4. 

### 3.5. Rh1 Inhibits Ang II-Increased KLF4 Activity and Phenotypic Switching in VSMCs via the ERK1/2/p90RSK Signaling Pathway 

**Figure 5:**
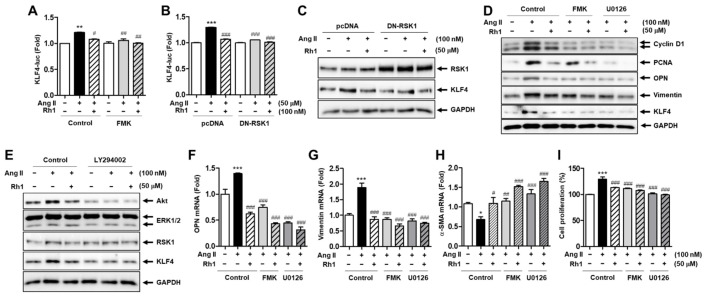
Rh1 inhibits Ang II-induced increase in the KLF4 promoter activity and phenotypic switching of VSMCs via the ERK1/2/p90RSK signaling pathway. (**A**) After being transfected with KLF4-Luc and Renilla-Luc, RASMCs were incubated with 20 µM FMK and/or 50 µM Rh1 for 1 h and then treated with Ang II (100 nM) for 12 h. KLF4-Luc promoter activity was evaluated after performing normalization with the Renilla luciferase activity. (**B**) The cells were transfected with control plasmid DNA (pcDNA) or dominant negative RSK1 (DN-RSK1) for 12 h and then transfected with KLF4-Luc and Renilla-Luc. Next, the cells were pretreated with 50 µM Rh1 for 1 h and then treated with Ang II (100 nM) for 12 h. Subsequently, KLF4-Luc promoter activity was evaluated after performing normalization with the Renilla luciferase activity. (**C**) After the cells were transfected with pcDNA or DN-RSK1, they were preincubated with 50 µM Rh1 for 3 h and then treated with Ang II (100 nM) for 24 h. (**D**) The cells were preincubated with 20 µM FMK or 10 µM U0126 and/or 50 µM Rh1 for 3 h and then stimulated with Ang II (100 nM) for 24 h. (**E**) Cells were preincubated with 5 µM LY294002 and/or 50 µM Rh1 for 3 h and then exposed to Ang II (100 nM) for 24 h. (**C**–**E**) Western blot was used to analyze the levels of indicated proteins in the whole-cell lysates. (**F**–**H**) RASMCs were preincubated with 50 µM Rh1 in the presence or absence of 20 µM FMK or 10 µM U0126 for 1 h, before undergoing stimulation with Ang II (100 nM) for 12 h. The mRNA expression levels of OPN, vimentin, and α-SMA were evaluated using qPCR analysis. (**I**) The cell proliferation was examined by SRB assay. (**A**,**B**,**F**–**I**) Results are represented as the mean ± SEM (*n* = 4), * *p* < 0.05, ** *p* < 0.01, and *** *p* < 0.001 compared with the control. # *p* < 0.05, ## *p* < 0.01, and ### *p* < 0.001 compared with the Ang II-treated sample.

Because Rh1 has the ability to inactivate ERL1/2 and p90RSK as well as suppress KLF4 protein expression induced by Ang II, we next identified the correlation between KLF4 and ERK1/2/p90RSK in the effect of Rh1 in Ang II-stimulated VSMCs. The inhibition of p90RSK using FMK slightly enhanced the effects of Rh1 on both the transcriptional activity and protein expression of KLF4. Subsequently, the combination of Rh1 and FMK completely abolished the impact of Ang II on these activities (Figure 5A,D). Consistently, the inactivation of p90RSK by overexpressing dominant negative RSK1 led to the same results in terms of the KLF4 protein expression and promoter activity (Figure 5B,C). 

In addition, the inhibition of p90RSK and ERK1/2 signaling suppressed the Ang II-increased KLF4 protein expression and further enhanced the inhibitory effect of Rh1 on the protein expression of this phenotypic switching regulator and synthetic markers, including OPN and vimentin (Figure 5D). Consistently, a similar pattern was observed in the mRNA expression of OPN and vimentin (Figure 5F,G). In contrast, the inhibitors of p90RSK and ERK1/2 recovered the Ang II-induced downregulation in mRNA expression of the contractile marker, α-SMA, and further increased the effect of Rh1 on the mRNA level of α-SMA (Figure 5H). In addition, the phenotypic switching of VSMCs is closely linked to cell proliferation [31]. Interestingly, the inhibitory effect of Rh1 on Ang II-induced RASMC proliferation was also associated with the inhibition of the ERK1/2/p90RSK pathway (Figure 5I). Consistent with our previous findings, which demonstrated the critical role of p90RSK in Akt activation in Ang II-stimulated VSMCs [9], the current results showed that the inhibition of Akt by LY294002 also inhibited the KLF4 protein expression induced by Ang II (Figure 5E). These results confirm that KLF4 is downstream of the Ang II-induced ERK1/2/p90RSK pathway in RASMCs and that Rh1 inhibits KLF4 to regulate VSMC phenotypic switching by inactivating the ERK1/2/p90RSK signaling pathway.

### 3.6. Rh1 Inhibits the Ang II-Induced Migration of RASMCs via the ERK1/2/p90RSK Signaling Pathway

**Figure 6:**
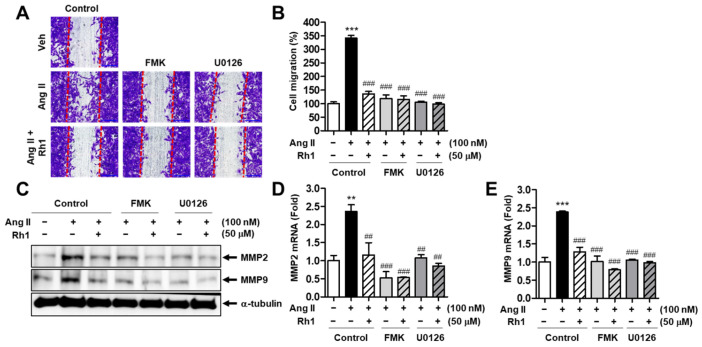
Rh1 inhibits VSMC migration promoted by Ang II via the ERK1/2/p90RSK signaling pathway. (**A**–**C**) The cells were pretreated with 50 µM Rh1 in the presence or absence of 20 µM FMK or 10 µM U0126 for 3 h, before undergoing stimulation with Ang II (100 nM) for 24 h. (**A**,**B**) The wound-healing assay was applied to examine the cell migration. The red lines indicate a clear zone after scratching (0 h). (**B**) The quantification of cell migration was carried out by Image J software. (**C**) Western blot analysis was used to analyze the levels of the indicated proteins in the cells. (**D**,**E**) RASMCs were preincubated with 50 µM Rh1 in the presence or absence of 20 µM FMK or 10 µM U0126 for 1 h, before 12 h of exposure to 100 nM Ang II. The mRNA levels of MMP2 and MMP9 were evaluated using qPCR analysis. (**B**,**D**,**E**) Results are presented as means ± SEM (*n* = 4), ** *p* < 0.01 and *** *p* < 0.001 compared with the control. ## *p* < 0.01 and ### *p* < 0.001 compared with the Ang II-treated sample.

Because Rh1 displayed an inhibitory effect on ERK1/2 and p90RSK phosphorylation, FMK and U0126 were used to confirm the involvement of the inhibition of the ERK1/2/p90RSK pathway in Rh1 effects on cell migration. First, as revealed by the wound-healing assay, the blocking of p90RSK activation by FMK and abolishing of ERK1/2 kinase activity by U0126 enhanced the inhibitory effect of Rh1 on the cell migration induced by Ang II (Figure 6A,B). Ang II increased the cell migration to 341.4 ± 10.00% (*p* < 0.001) as compared with the control group, and the combination of FMK and Rh1 or U0126 and Rh1 decreased the cell migration induced by Ang II to 115.7 ± 13.09% (*p* < 0.001) or 98.57 ± 4.95% (*p* < 0.001), respectively (Figure 6A,B). In correlation with that, these inhibitors also further decreased the inhibitory effects of Rh1 on the elevated levels of both the protein expression and mRNA expression of migration markers, MMP2 and MMP9, induced by Ang II (Figure 6C–E). Collectively, these data indicate that Rh1 inhibits Ang II-induced cell migration through inhibition of the ERK1/2/p90RSK pathway. 

### 3.7. Rh1 Inhibits Ang II-Induced Superoxide Production in RASMCs

**Figure 7:**
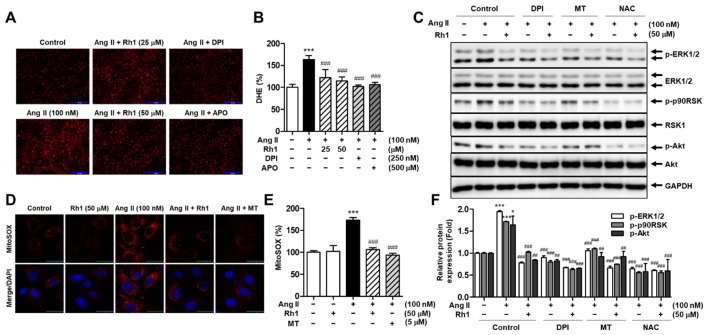
Rh1 suppresses reactive oxygen species (ROS) production in Ang II-stimulated VSMCs. (**A**,**B**) RASMCs were pretreated with Rh1 (25 and 50 µM), 250 nM DPI, or 500 µM APO for 1 h, and then stimulated with Ang II for an additional 12 h. The intracellular superoxide levels were measured by DHE assay. (**D**,**E**) RASMCs were pretreated with 50 µM Rh1 or 5 µM Mito-TEMPO (MT) for 1 h and then exposed to Ang II (100 nM) for 1 h. Mitochondrial superoxide generation was examined using MitoSOX staining. (**C**,**F**) RASMCs were pretreated with 50 µM Rh1 in the presence or absence of DPI, MT, or NAC for 1 h, before 1 h of exposure to 100 nM Ang II. Western blot was used to analyze the levels of indicated proteins in the whole-cell lysates. (**B**,**E**,**F**) Results are presented as means ± SEM (*n* = 4), * *p* < 0.05 and *** *p* < 0.001 compared with the control. ## *p* < 0.01 and ### *p* < 0.001 compared with the Ang II-treated sample. APO: apocynin, DPI: diphenyleneiodonium, MT: Mito-TEMPO, NAC: N-acetylcysteine, DHE: dihydroethidium.

In Ang II-stimulated VSMCs, superoxide anions activate the MAPK pathway [12]. Therefore, the effect of Rh1 on superoxide production stimulated by Ang II was evaluated in RASMCs. We observed that Rh1 treatment remarkably reduced the intracellular superoxide production increased by Ang II, as evidenced by DHE staining (Figure 7A,B). DPI and APO, which are NOX inhibitors, were used as negative controls. The inhibitory effect of 50 µM Rh1 on the intracellular superoxide production (% of decrease vs. Ang II group: 48.5 ± 3.66%, *p* < 0.001) was relatively similar to that of 250 nM DPI (% of decrease vs. Ang II group: 61.8 ± 3.63%, *p* < 0.001) and that of 500 µM APO (% of decrease vs. Ang II group: 57.1 ± 4.76%, *p* < 0.001) (Figure 7A,B). Similarly, the Rh1 treatment inhibited Ang II-induced mitochondrial superoxide production (Figure 7D,E). Ang II increased mitochondrial superoxide production to 173.00 ± 5.75% (*p* < 0.001) compared with the control group; meanwhile, 50 µM Rh1 reversed the effect of Ang II to 106.10 ± 2.38% (*p* < 0.001). Mito-TEMPO (MT) was used as a negative control for assessing the mitochondrial superoxide production. 

Additionally, to confirm the role of ROS production in the effect of Rh1 on Ang II-stimulated activation of the ERK1/2/p90RSK pathway, RASMCs were pretreated with various inhibitors of ROS, including a non-selective NOX inhibitor, DPI, a mitochondrial ROS inhibitor, MT, and an ROS scavenger, N-acetylcysteine (NAC), before treatment with 100 nM Ang II for 1 h. The results showed that all three inhibitors further enhanced the inhibitory effect of Rh1 on the activation of ERK1/2, p90RSK, and Akt; NAC showed the highest inhibitory effect among the three ROS inhibitors (Figure 7C,F). These data indicate that Rh1 inhibits the ERK1/2/p90RSK signaling pathway through the inhibition of the ROS production generated from NOXs and mitochondria in Ang II-stimulated RASMCs. 

### 3.8. Rh1 Suppresses Ang II-Stimulated RASMC Migration through Inhibition of ROS 

**Figure 8:**
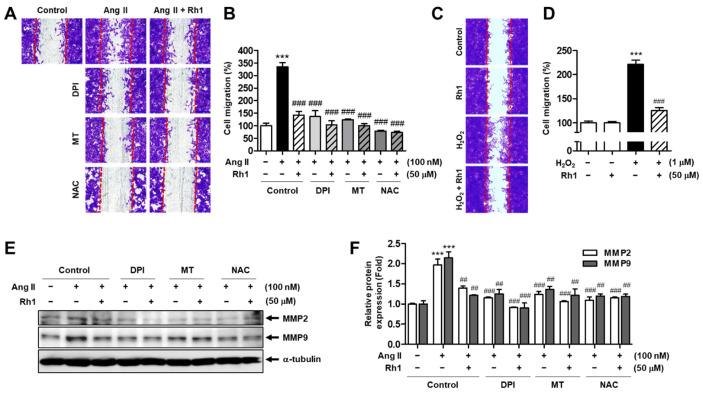
Rh1 suppresses the cell migration stimulated by Ang II through the inhibition of ROS production. (**A**,**B**) RASMCs were pretreated with 50 µM Rh1 in the presence or absence of the indicated inhibitors for 3 h before exposure to Ang II (100 nM) for an additional 24 h. (**A**) The wound-healing assay was performed to examine the cell migration. The red lines indicate a clear zone after scratching (0 h). (**B**) The quantification of cell migration was carried out by Image J software. (**C**,**D**) The cells were preincubated with 50 µM Rh1 for 3 h before treatment with 1 µM H_2_O_2_ for 24 h. The cell migration was then evaluated by the wound-healing assay. The red lines indicate a clear zone after scratching (0 h). (**E**,**F**) The cells were pretreated with 50 µM Rh1 in the presence or absence of the indicated inhibitors for 3 h before exposure to Ang II (100 nM) for an additional 24 h. Western blot analysis was used to analyze the levels of the indicated proteins in the whole-cell lysates. (**B**,**D**,**F**) Results are represented as means ± SEM (*n* = 4), *** *p* < 0.001 compared with the control, ## *p* < 0.01 and ### *p* < 0.001 compared with the Ang II-treated sample. DPI: diphenyleneiodonium, MT: Mito-TEMPO, NAC: N-acetylcysteine.

Next, we examined whether Rh1 suppressed the migration of RASMCs induced by Ang II via ROS inhibition. RASMCs were preincubated with DPI, MT, or NAC in the presence or absence of Rh1 for 3 h, before being administered treatment with Ang II for 24 h. As demonstrated by the results of the wound-healing assay, the ROS inhibitors further enhanced the inhibitory effect of Rh1 on the cell migration induced by Ang II (Figure 8A,B). However, there were no significant differences in the inhibitory effect between the pretreatment with each inhibitor alone and the combination of each inhibitor and Rh1 (Figure 8A,B). To investigate the role of Rh1 in VSMCs stimulated with exogenous ROS, the cells were pretreated with Rh1 and then exposed to hydrogen peroxide (H_2_O_2_). As a result, increased exogenous ROS augmented VSMC migration to 221.0 ± 8.96% (*p* < 0.001), whereas the pretreatment with Rh1 rescued these effects to 125.2 ± 5.92% (*p* < 0.001) (Figure 8C,D). Interestingly, protein expression MMP2 and MMP9 induced by Ang II were consistently regulated by the ROS inhibitors (Figure 8E,F). These data imply that the inhibition of ROS production plays a key role in the inhibitory effect of Rh1 on Ang II-induced VSMC migration. 

### 3.9. Rh1 Suppresses Ang II-Stimulated RASMC Proliferation through Inhibition of ROS 

**Figure 9:**
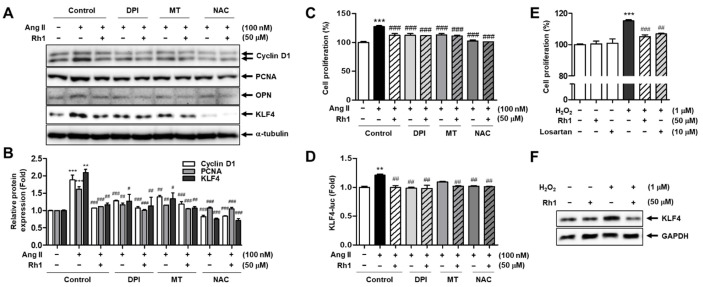
Rh1 suppresses the cell proliferation induced by Ang II through the inhibition of ROS production. (**A**–**C**) RASMCs were pretreated with 50 µM Rh1 in the presence or absence of 250 nM DPI, 5 µM MT, or 5 mM NAC for 3 h, before exposure to Ang II for an additional 24 h. Western blot analysis was used to analyze the levels of indicated proteins in the whole-cell lysates (**A**,**B**). The cell proliferation was examined by SRB assay (**C**). (**D**) After transfection with KLF4-Luc and Renilla-Luc, the cells were pretreated with 50 µM Rh1 in the presence or absence of DPI, MT, or NAC for 3 h, before exposure to Ang II for an additional 12 h. KLF4-Luc promoter activity was calculated by performing normalization with Renilla luciferase activity. (**E**,**F**) The cells were preincubated with 50 µM Rh1 or 10 µM losartan before being administered treatment with 1 µM H_2_O_2_. The cell proliferation was evaluated by the SRB assay (**E**). Western blot analysis was used to analyze the levels of indicated proteins in the whole-cell lysates (**F**). (**B**–**E**) Results are represented as the mean ± SEM (*n* = 4), ** *p* < 0.01 and *** *p* < 0.001 compared with the control, # *p* < 0.05, ## *p* < 0.01, and ### *p* < 0.001 compared with the Ang II-treated sample. DPI: diphenyleneiodonium, MT: Mito-TEMPO, NAC: N-acetylcysteine.

Similarly, the importance of ROS inhibition in suppressing Ang II-induced RASMC proliferation following Rh1 pretreatment was investigated. The results of the SRB assay revealed that Ang II increased cell proliferation to 127.3 ± 1.82% (*p* < 0.001), and Rh1 declined the Ang II-induced cell proliferation to 112.2 ± 1.92% (*p* < 0.001), the same degree as DPI alone (112.3 ± 1.82%), MT alone (113.2 ± 2.13%), or the combination of Rh1 and DPI or MT (111.7 ± 0.32% or 111.0 ± 1.29%, respectively) (Figure 9C). Meanwhile, NAC further enhanced the inhibitory effect of Rh1 to 101.1 ± 0.24% (*p* < 0.001); however, there were no significant differences between the effect on cell proliferation by NAC and those by the combination of Rh1 and NAC (Figure 9C). In addition, the protein levels of cyclin D1, PCNA, and OPN were shown to have the same pattern from the cell proliferation results (Figure 9A,B). Consistently, the effect of Rh1 on Ang II-increased KLF4 protein expression and promoter activity was found to be associated with the ROS inhibition (Figure 9A,B,D). In addition, increasing exogenous ROS by treating the cells with H_2_O_2_ upregulated the KLF4 protein expression and increased the VSMC proliferation, and these effects were suppressed by the Rh1 pretreatment (Figure 9E,F). These findings imply that the mechanism by which Rh1 suppresses the Ang II-induced proliferation of VSMCs involves the inhibition of ROS.

## 4. Discussion

Inflammation is known to be associated with atherosclerosis [33]. We previously revealed that Rh1 and Rg2 combination displayed anti-inflammatory activity in macrophages stimulated by lipopolysaccharides [16]. We also demonstrated that this combination treatment protected liver damages under septic condition through the Nrf2/ARE-mediated antioxidant signaling pathway [21]. In addition, Rh1 plays an important role in immune regulation, and possesses various cardiovascular benefits [24,25]. Therefore, we investigated whether Rh1 could inhibit Ang II-induced VSMC proliferation and migration, which are two major events associated with the progression of atherosclerosis.

The effects of Ang II are primarily manifested via two G-protein-coupled, seven-transmembrane-domain receptors, AT1R and AT2R [34]. These two receptors are associated with different signaling pathways and mediate different functions. In fact, AT1R activates growth-promoting pathways and mediates major Ang II effects, such as vasoconstriction, increased blood pressure, cardiac contractility, and cell proliferation [34]. On the other hand, AT2R is believed to induce opposing effects, namely vasodilatation, hypotension, antigrowth by apoptosis, anti-hypertrophic effects, and the possible inhibition of AT1R [34]. In the current study, we aimed to discover the agent that can regulate AT1R-mediated growth-promoting signaling pathway in VSMCs. 

We found that Rh1 suppressed the cell migration and proliferation induced by Ang II in a dose-dependent manner (Figure 1). MMPs regulate cell migration by degrading the extracellular matrix [35]. Here, Rh1 was shown to decrease the elevated protein expression of MMP2 and MMP9 in Ang II-stimulated RASMCs, which was consistent with the inhibitory effect of Rh1 on cell migration that was observed in the wound-healing assay (Figure 1). Mechanistically, the inhibition of ERK1/2 by U0126 and inhibition of p90RSK by FMK inhibited both the increased protein expression and mRNA levels of MMP2 and MMP9 induced by Ang II (Figure 6C–E), suggesting that MMP2 and MMP9 are downstream of the ERK1/2/p90RSK pathway. In addition, cyclin D1 and PCNA are key regulators of VSMC proliferation [36]. In agreement with this, Rh1 decreased the cell viability as well as cyclin D1 and PCNA protein expression in Ang II-stimulated RASMCs (Figure 2A–C). Furthermore, VSMC phenotypic switching, characterized by an increase in synthetic markers and a decrease in contractile proteins, is associated with cell proliferation and migration [31]. In Ang II-stimulated VSMCs, in addition to decreasing cyclin D1, PCNA, MMP2, and MMP9, Rh1 pretreatment also caused a decrease in the protein expression of OPN and vimentin, synthetic markers, along with an increase in the protein levels of α-SMA, a VSMC-specific contractile marker (Figure 2). This is consistent with previous findings, in which targeting these molecules by various natural compounds, such as luteolin, apamin, brazilin, crocetin, piperine, and resveratrol, inhibited the VSMC proliferation and migration [37,38]. Luteolin inhibited VSMC proliferation and migration by downregulating the expression of cyclin D1, PCNA, MMP2, and MMP9 [38]. Gossypetin and thymoquinone inhibited VSMC migration by decreasing MMP9 expression [14,39].

The MAPK pathway plays a paramount role in the regulation of VSMC proliferation and migration, and targeting this pathway is a therapeutic strategy for treating atherosclerosis [9]. Furthermore, Ang II promotes VSMC proliferation and migration by activating ERK1/2 and its downstream targets [40]. In addition to natural compounds, various agents, such as exendin-4, a glucagon-like peptide-1 receptor agonist, and rosuvastatin, a selective inhibitor of hydroxymethylglutaryl coenzyme A reductase, have also been demonstrated to inhibit VSMC proliferation and migration through inactivating the MAPK pathway [41,42]. We observed that Rh1 inhibited the phosphorylation of ERK1/2 and its downstream, p90RSK (Figure 3A,B). In addition, we have previously demonstrated that p90RSK activation is critical to Akt phosphorylation in Ang II-stimulated VSMCs [9]. In line with this, a similar observation was made in the present study, in which Rh1 pretreatment resulted in the inactivation of ERK1/2 and p90RSK, along with Akt deactivation, in Ang II-activated RASMCs (Figure 3A,B). Furthermore, we previously found that the inhibition of Akt by LY294002 inhibited the cell proliferation and migration induced by Ang II, suggesting a role for Akt in promoting VSMC proliferation and migration [9]. Moreover, the inhibition of ERK1/2 activation as well as the inhibition of p90RSK kinase activity enhanced the inhibitory effects of Rh1 on Ang II-induced RASMC migration and proliferation and the protein expression of related molecules, including MMP2, MMP9, PCNA, cyclin D1, OPN, vimentin, and KLF4 (Figure 4, Figure 5 and Figure 6). These data suggest that the inhibitory effect of Rh1 on RASMC proliferation and migration is associated with the inhibition of the ERK1/2/p90RSK pathway. 

ROS have been found to exert detrimental impacts in the vasculature [43]. In particular, ROS promote VSMC proliferation and migration, facilitating the development of cardiovascular diseases [44]. ROS are derived from various sources, including NOXs, mitochondria, xanthine oxidase, lipoxygenase, and the uncoupling nitric oxide synthase [45]. Among the types of ROS, superoxide anions have been found to activate the ERK1/2 pathway in VSMCs [12]. Therefore, we focused on the relationship between the inhibitory effects of Rh1 and superoxide anions in Ang II-stimulated VSMCs. NOXs and mitochondria are important sources of superoxide anions [46,47,48]. The incubation with Rh1 decreased both the intracellular and mitochondrial superoxide production elevated by Ang II (Figure 7). In addition, ROS inhibition by DPI, MT, or NAC not only suppressed the Ang II-induced phosphorylation of ERK1/2, p90RSK, and Akt, but also further enhanced the inhibitory effect of Rh1 on the activation of these kinases (Figure 7C). Similar data were observed when evaluating the role of ROS inhibition on cell migration and proliferation as well as on the protein levels of MMP2, MMP9, cyclin D1, PCNA, OPN, and KLF4 (Figure 8 and Figure 9). Collectively, these findings indicate that the anti-migration and anti-proliferation effects of Rh1 on Ang II-stimulated RASMCs are attributed to the suppression of the ROS-mediated ERK1/2/p90RSK signaling pathway. Interestingly, in terms of the ROS production, Rh1 treatment resulted in opposite responses in MCF-7 cells and VSMCs. The ROS production was inhibited by Rh1 treatment in Ang II-stimulated RASMCs; however, Rh1 promoted an excessive production of ROS in MCF-7 breast cancer cells, killing these cells, which was demonstrated in our previous report [17].

KLF4 has been reported to regulate the phenotypic switching of VSMCs under the stimulation of several factors, including PDGF and oxidized phospholipids, as well as in atherosclerotic lesions [13,32,49,50]. Indeed, the depletion of KLF4 was found to inhibit the downregulation of contractile markers, such as α-SMA and calponin, and the upregulation of synthetic markers, including vimentin and OPN [32,50]. Consistent with these findings, we demonstrated that Ang II induced the increase in KLF4 protein expression and promoter activity, along with the downregulation of α-SMA expression and upregulation of OPN and vimentin expression, whereas Rh1 preincubation reversed these effects (Figure 2 and Figure 4). These findings indicate that Rh1 can inhibit VSMC phenotypic switching through downregulation of KLF4, and subsequently suppress VSMC proliferation and migration. Notably, KLF4 was demonstrated to be a downstream effector of the ERK1/2 signaling pathway in Ang II-stimulated VSMCs, and Rh1 inhibited KLF4 via the inhibition of the ERK1/2 pathway (Figure 5). 

In addition, several microRNAs (miRs), including miR-93 and miR-145, have been reported to be associated with VSMC proliferation and migration [51,52]. In here, miR-93 inhibitor decreased the expression of MMP2 and cyclin D1 through regulating the ERK1/2 pathway [52]. The downregulation of miR-145 is associated with the increase of KLF4 activity in Ang II-stimulated VSMCs [51]. Therefore, if the ginsenoside Rh1 affects these miRs in stimulated VSMCs, it is worth investigating in future studies.

There are several studies that have reported the effect of Rh1 on cardiovascular effects. Rh1 had a beneficial effect on cardiomyocytes by reducing the open-state probabilities of three types of calcium channels, including B, L, and T types at a concentration of 60 µM [53]. In another study, Rh1 at 25 µM showed a significant attenuation of monocyte adhesion of HUVECs stimulated by TNF-α at 10 ng/mL without any cytotoxicity [54]. Consistent with similar effective Rh1 concentrations seen in other group studies, our results showed that Rh1 at 25~50 µM did not induce cytotoxicity, but had a remarkable inhibitory effects on Ang II-induced cell migration and proliferation. Ginsenosides have been reported to have low oral bioavailability. However, its absorption in vivo can be improved and enhanced by formulation strategies, such as nanoparticle drug delivery systems, liposome drug delivery systems, emulsion delivery systems, and so on [55,56]. With the advanced drug delivery systems, drugs can be controlled and released to reach a desired concentration at targeted tissues. Therefore, Rh1 with these doses can be appropriate to develop a promising therapeutic for cardiovascular diseases.

The present study showed that Rh1 inhibited Ang II-induced VSMC proliferation and migration by suppressing the intracellular and mitochondrial ROS-mediated ERK1/2/p90RSK/KLF4 pathway. Rh1 suppressed Ang II-induced RASMC migration and proliferation and also reduced the protein levels of cell migration-related and cell proliferation-related biomarkers, such as MMP2, MMP9, PCNA, and cyclin D1. Rh1 treatment also prevented phenotypic switching to synthetic phenotype under the stimulation of Ang II, as the ginsenoside treatment reversed the changes in α-SMA, vimentin, and OPN protein levels induced by Ang II. Notably, Rh1 treatment abolished Ang II-increased the protein expression and transcriptional activity of KLF4, a key regulator of VSMC phenotypic switching. Mechanistically, Rh1 treatment resulted in deactivation of ERK1/2 and its downstream effector, p90RSK, which was accompanied by a decrease in NOXs and mitochondria-generated ROS levels in Ang II-stimulated RASMCs. These results imply that Rh1 had an inhibitory effect on the migration and proliferation of VSMCs induced by Ang II, and that Rh1 is a promising therapeutic agent for the treatment of vascular diseases, including atherosclerosis and restenosis.

## 5. Conclusions

In conclusion, the current study demonstrated that Rh1 inhibited the Ang II-induced phenotypic switching, as well as the proliferation and migration, of VSMCs. This inhibitory effect results from the suppression of KLF4 transcriptional activity through the inactivation of the ROS-mediated ERK1/2/p90RSK signaling pathway in Ang II-stimulated VSMCs. Therefore, Rh1 may serve as a potential therapeutic agent for the treatment of vascular diseases.

## Data Availability

Data is contained within the article and Supplementary Materials.

## Supplementary Materials

The following supporting information can be downloaded at: https://www.mdpi.com/article/10.3390/antiox11040643/s1, Figure S1: α-smooth muscle actin (α-SMA) expression in rat aortic smooth muscle cells at different passages (7, 10, and 15). File S1: Original Western Blot figures.
