# Supplementary material for: Ginsenoside Rh1 Inhibits Angiotensin II-Induced Vascular Smooth Muscle Cell Migration and Proliferation through Suppression of the ROS-Mediated ERK1/2/p90RSK/KLF4 Signaling Pathway
Source: Antioxidants (Basel). 2022 Mar 27;11(4):643. doi: 10.3390/antiox11040643 (PMC9030830; doi:10.3390/antiox11040643)
Supplement: Supplementary file 1 [file antioxidants-11-00643-s001.zip › Figure S1.pdf]

Supplementary figures

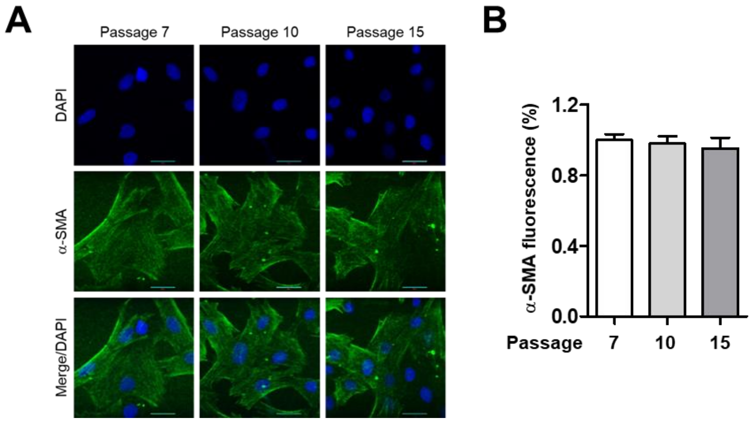

Figure S1. *α*-smooth muscle actin (*α*-SMA) expression in rat aortic smooth muscle cells at different passages (7, 10, and 15). The immunofluorescence analysis was performed after immunostaining with *α*-SMA antibody. DAPI was co-stained for indicating the nucleus. Representative photographs are shown at ×200 magnification (scale bar: 30 μm) (A). The relative fluorescent intensity of *α*-SMA was quantified by Image J software (B). Data are expressed as the mean ± SEM (*n* = 3).
