# Supplementary material for: Ginsenoside Rh1 Inhibits Angiotensin II-Induced Vascular Smooth Muscle Cell Migration and Proliferation through Suppression of the ROS-Mediated ERK1/2/p90RSK/KLF4 Signaling Pathway
Source: Antioxidants (Basel). 2022 Mar 27;11(4):643. doi: 10.3390/antiox11040643 (PMC9030830; doi:10.3390/antiox11040643)

Whole blot supplementary materials

Figure 1C

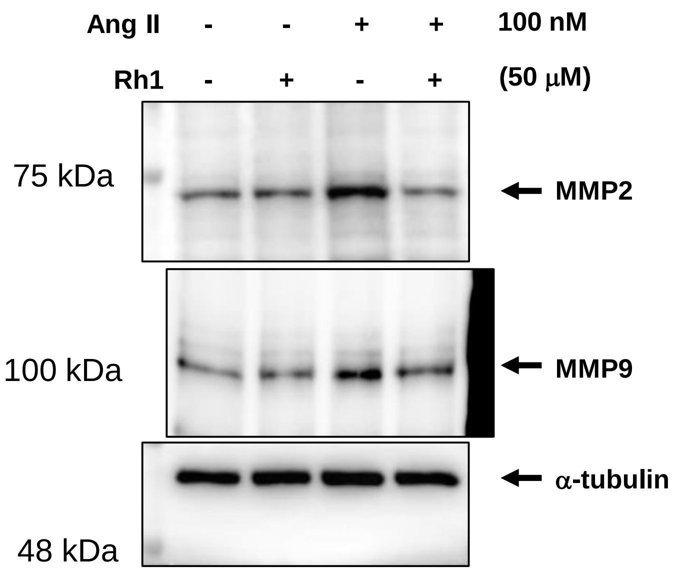

Figure 2B

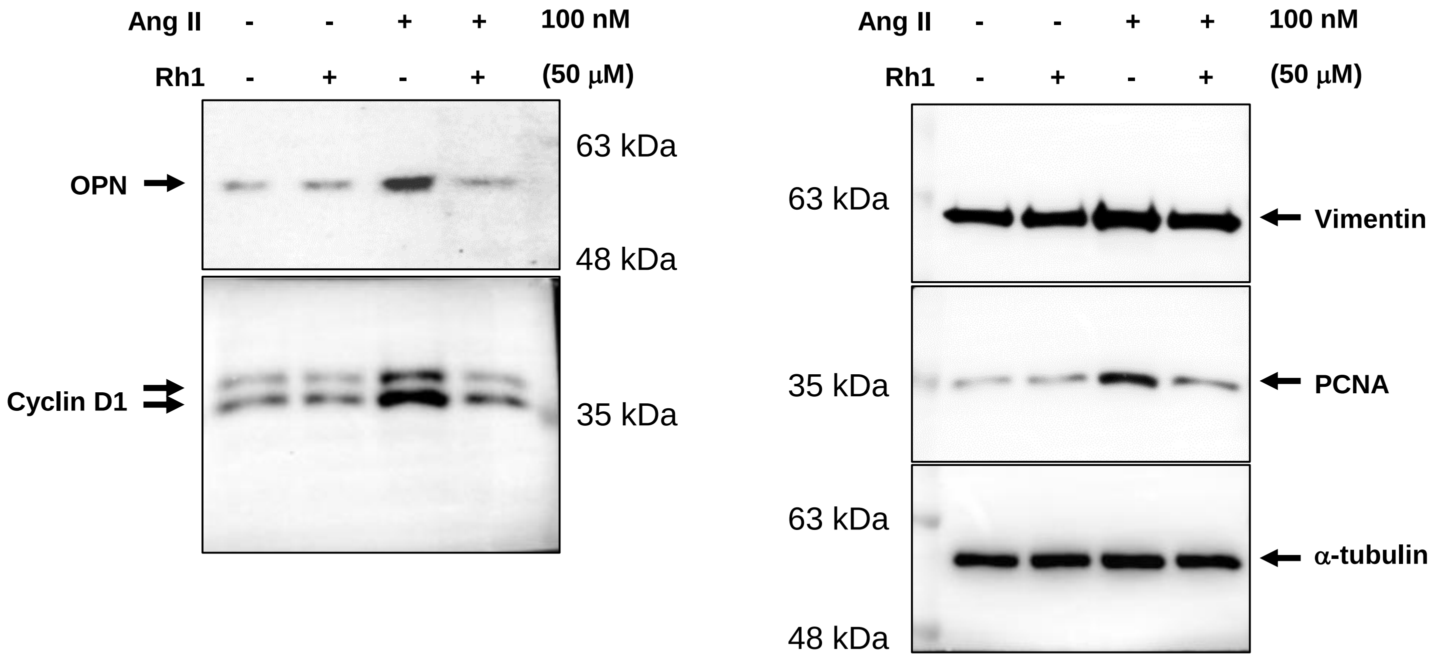

Figure 3A

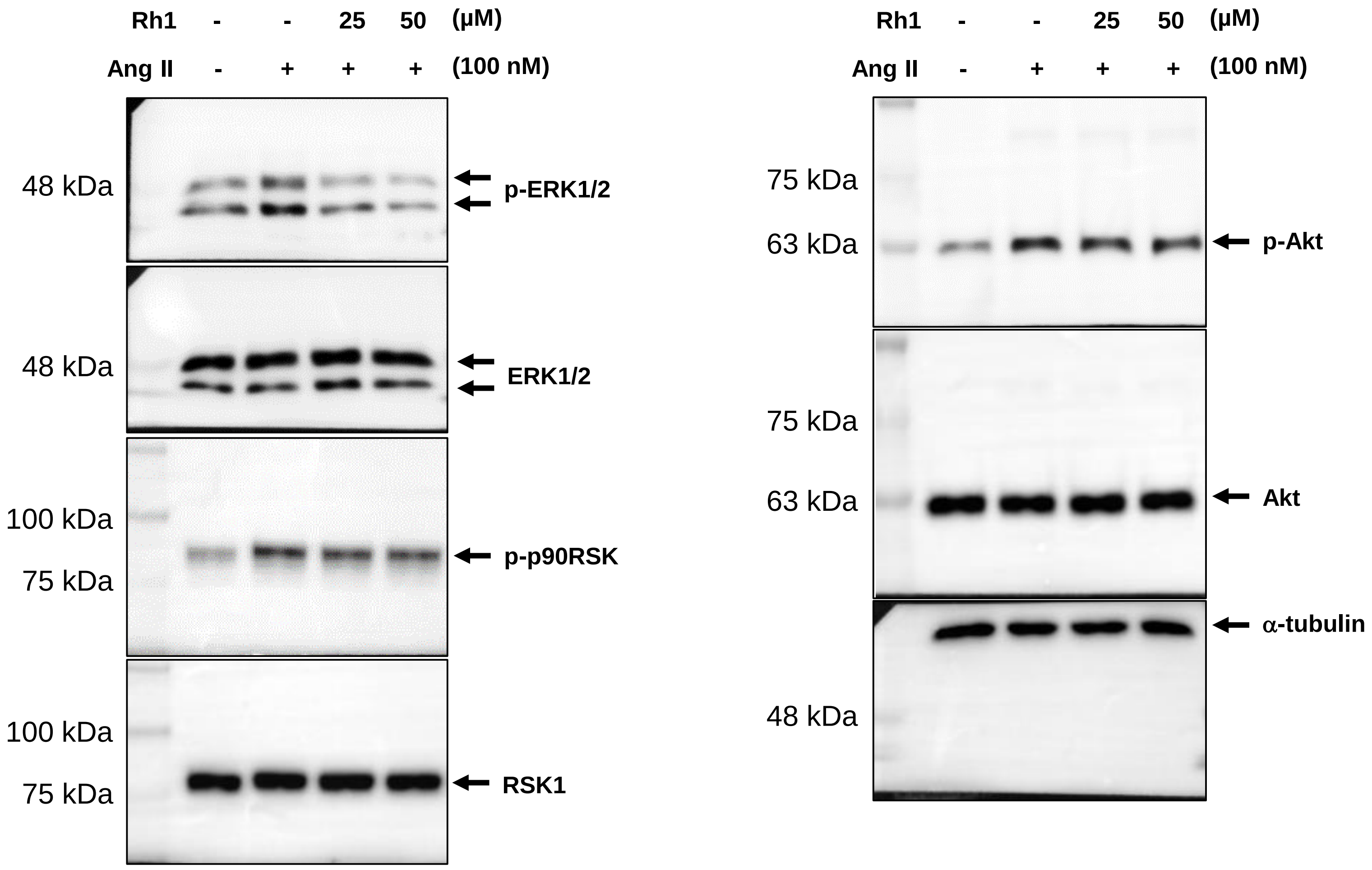

Rh1 - - 25 50 (μM)
Ang II - + + + (100 nM)
48 kDa
p-ERK1/2
48 kDa
ERK1/2
100 kDa
75 kDa
p-p90RSK
100 kDa
75 kDa
RSK1
Rh1 - - 25 50 (μM)
Ang II - + + + (100 nM)
75 kDa
63 kDa
p-Akt
75 kDa
63 kDa
Akt
α-tubulin
48 kDa

Figure 3C

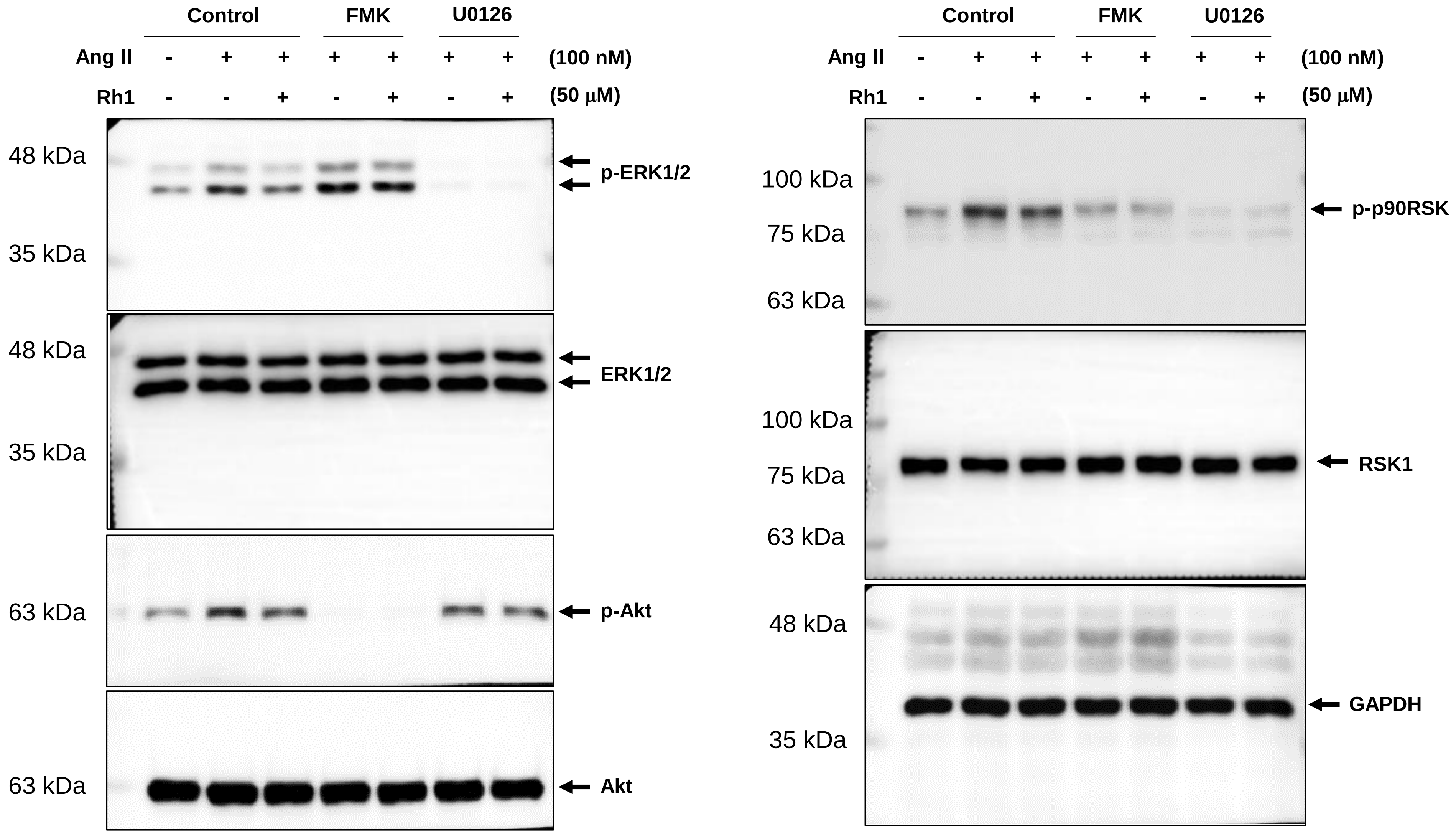

Control
FMK
U0126
Ang II - + + + + + + (100 nM)
Rh1 - - + - + - + (50 μM)
48 kDa
35 kDa
p-ERK1/2
48 kDa
35 kDa
ERK1/2
63 kDa
p-Akt
63 kDa
Akt
Control
FMK
U0126
Ang II - + + + + + + (100 nM)
Rh1 - - + - + - + (50 μM)
100 kDa
75 kDa
63 kDa
p-p90RSK
100 kDa
75 kDa
63 kDa
RSK1
48 kDa
35 kDa
GAPDH

Figure 4A

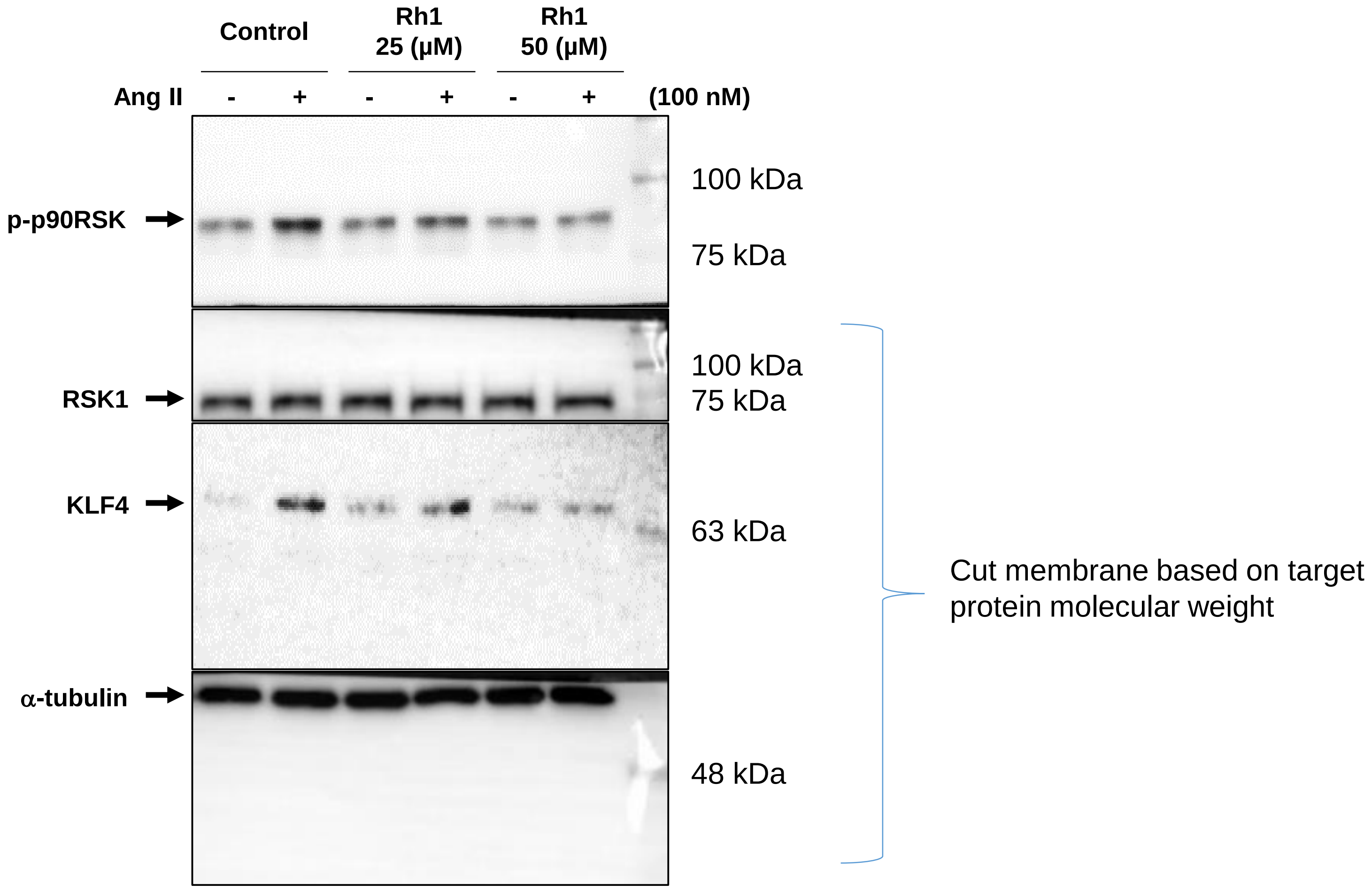
Control
Rh1
25 (μM)
Rh1
50 (μM)
Ang II - + - + - + (100 nM)
p-p90RSK
RSK1
KLF4
α-tubulin
100 kDa
75 kDa
100 kDa
75 kDa
63 kDa
48 kDa
Cut membrane based on target protein molecular weight

Figure 5C

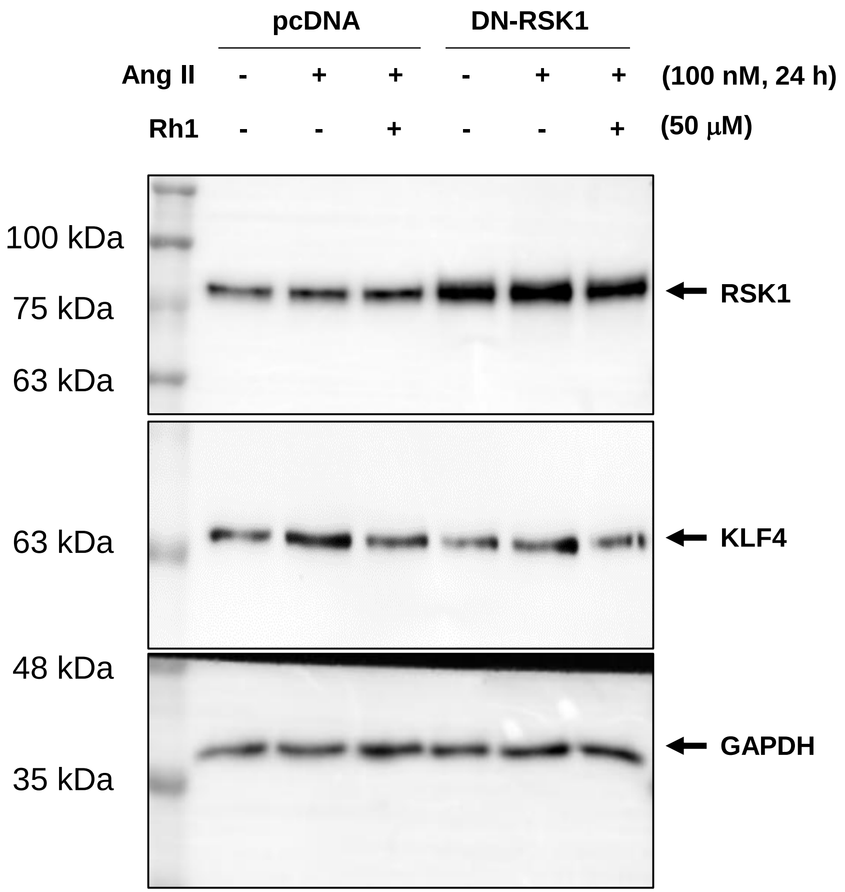

pcDNA
DN-RSK1
Ang II - + + - + + (100 nM, 24 h)
Rh1 - - + - - + (50 μM)
100 kDa
75 kDa
63 kDa
RSK1
63 kDa
KLF4
48 kDa
35 kDa
GAPDH

Figure 5D

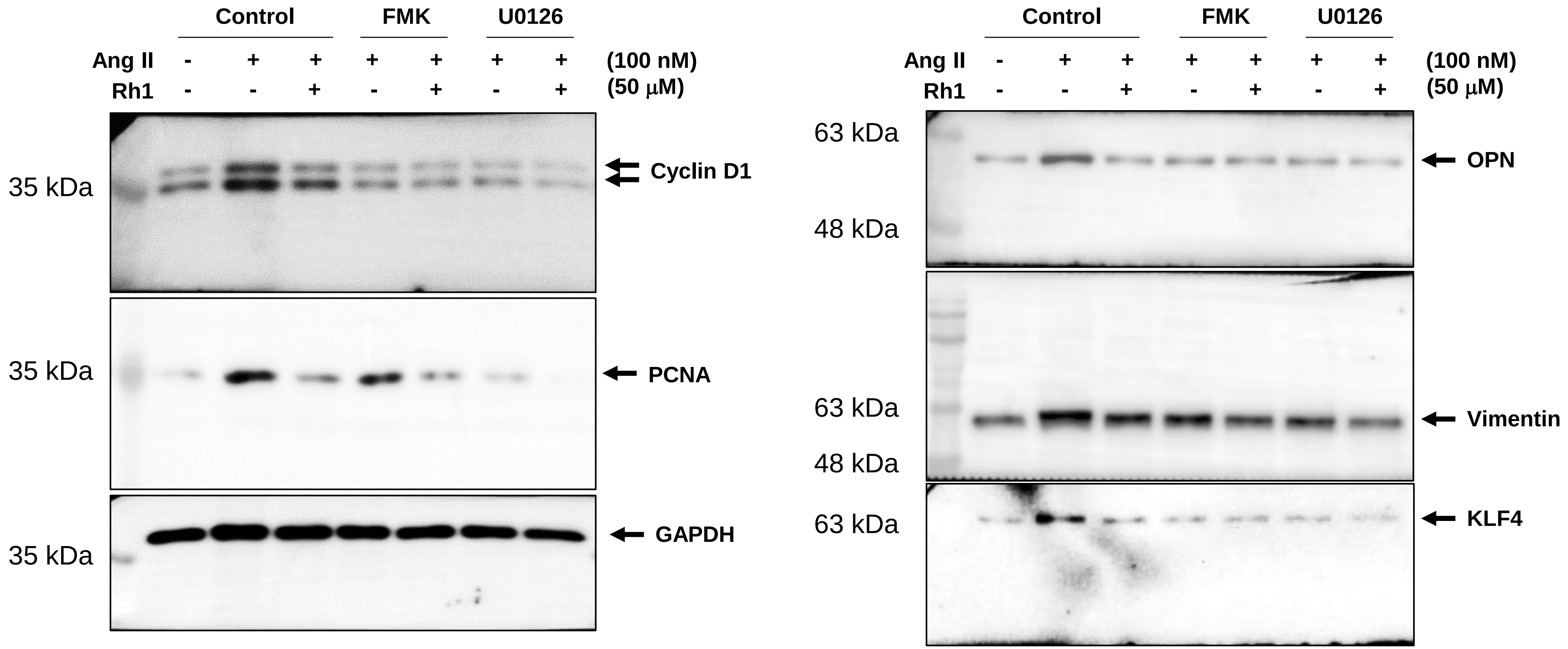
Control
FMK
U0126
Ang II - + + + + + + (100 nM)
Rh1 - - + - + - + (50 μM)
35 kDa
Cyclin D1
35 kDa
PCNA
35 kDa
GAPDH
Control
FMK
U0126
Ang II - + + + + + + (100 nM)
Rh1 - - + - + - + (50 μM)
63 kDa
48 kDa
OPN
63 kDa
48 kDa
Vimentin
63 kDa
KLF4

Figure 5E

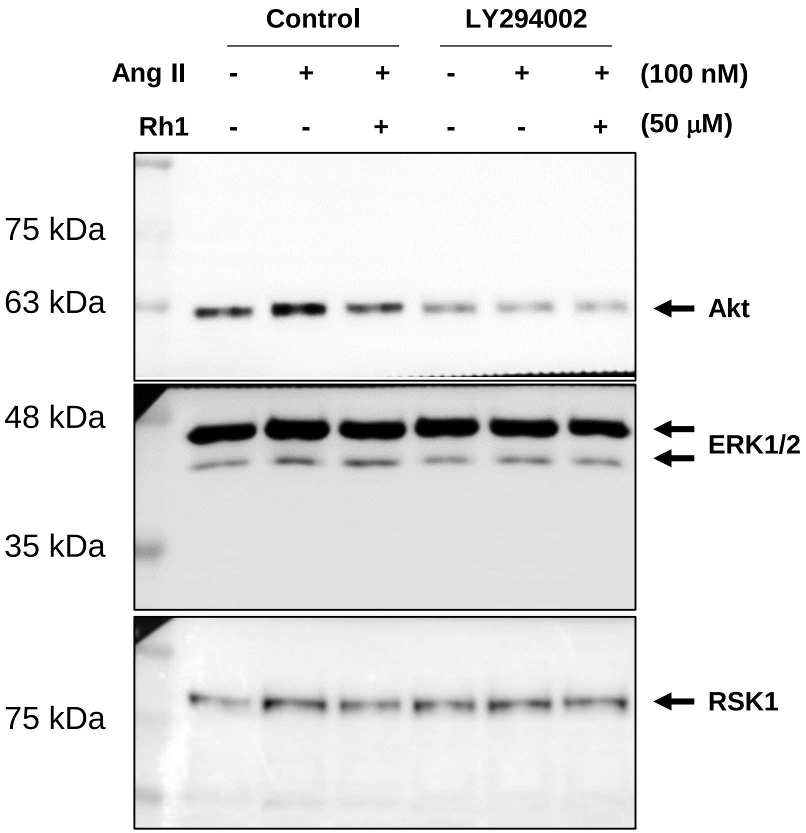
Control
LY294002
Ang II - + + - + + (100 nM)
Rh1 - - + - - + (50 μM)
75 kDa
63 kDa
Akt
48 kDa
ERK1/2
35 kDa
RSK1
75 kDa

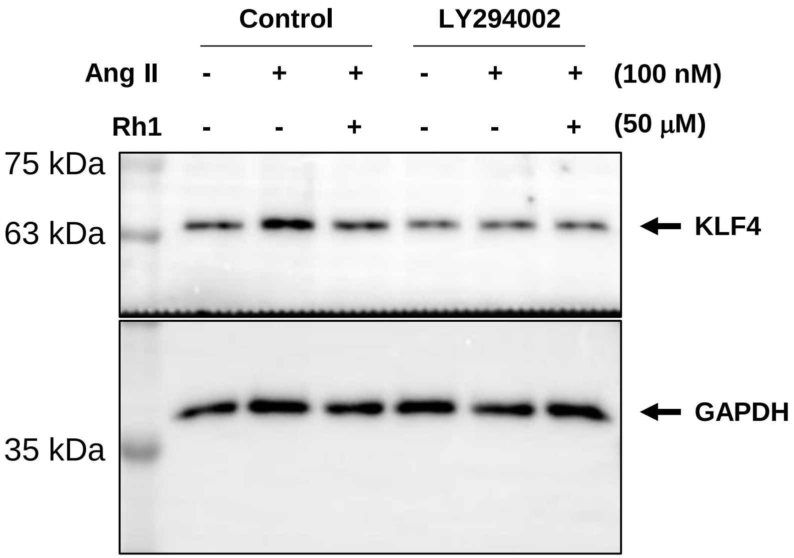
Control
LY294002
Ang II - + + - + + (100 nM)
Rh1 - - + - - + (50 μM)
75 kDa
63 kDa
KLF4
GAPDH
35 kDa

Figure 6C

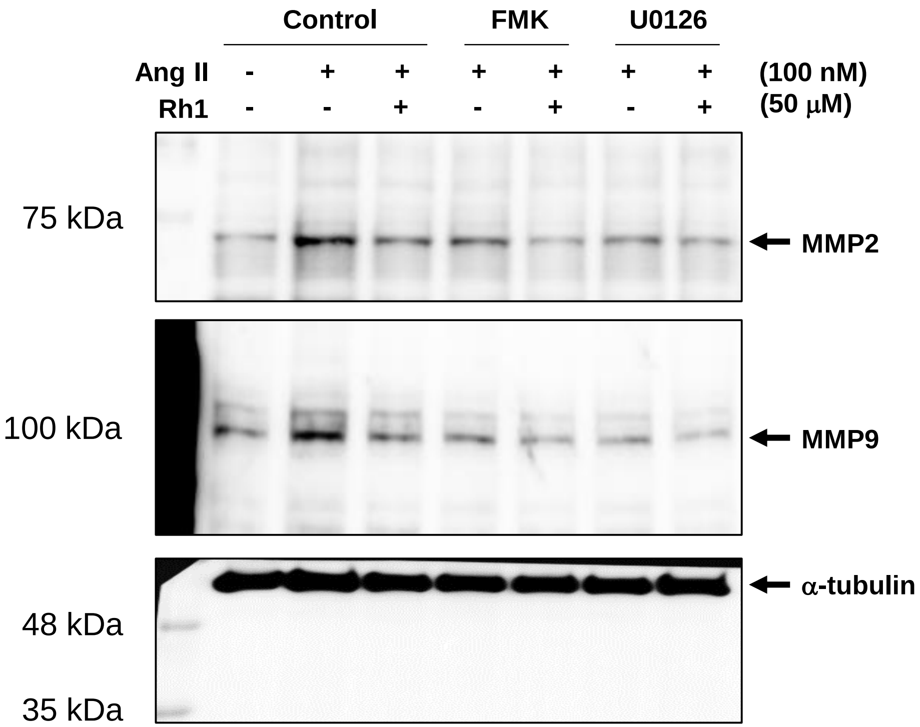

Control
FMK
U0126
Ang II - + + + + + + (100 nM)
Rh1 - - + - + - + (50 μM)
75 kDa
MMP2
100 kDa
MMP9
α-tubulin
48 kDa
35 kDa

Figure 7C

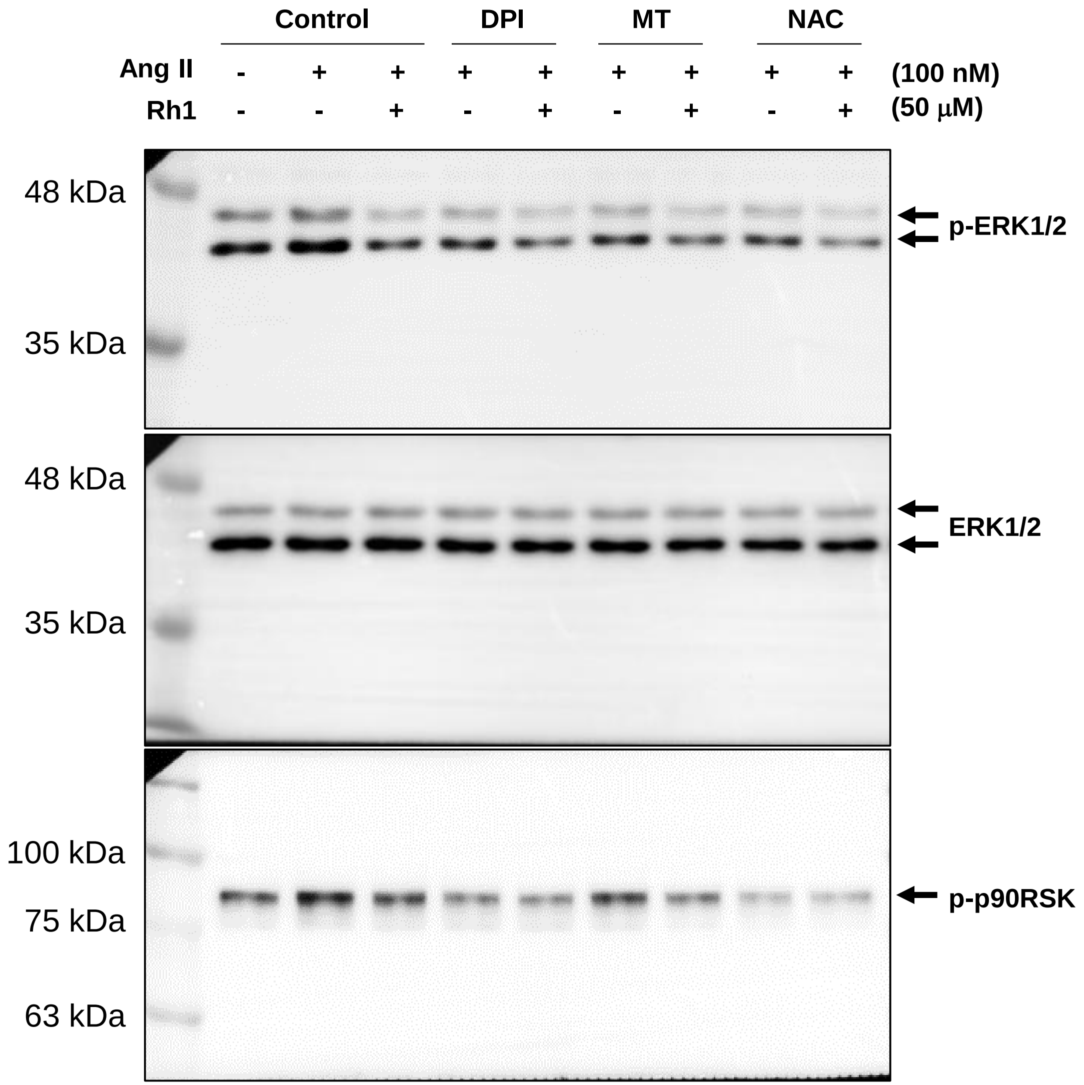

Control
DPI
MT
NAC
Ang II - + + + + + + + + (100 nM)
Rh1 - - + - + - + - + (50 μM)
48 kDa
35 kDa
p-ERK1/2
48 kDa
35 kDa
ERK1/2
100 kDa
75 kDa
63 kDa
p-p90RSK

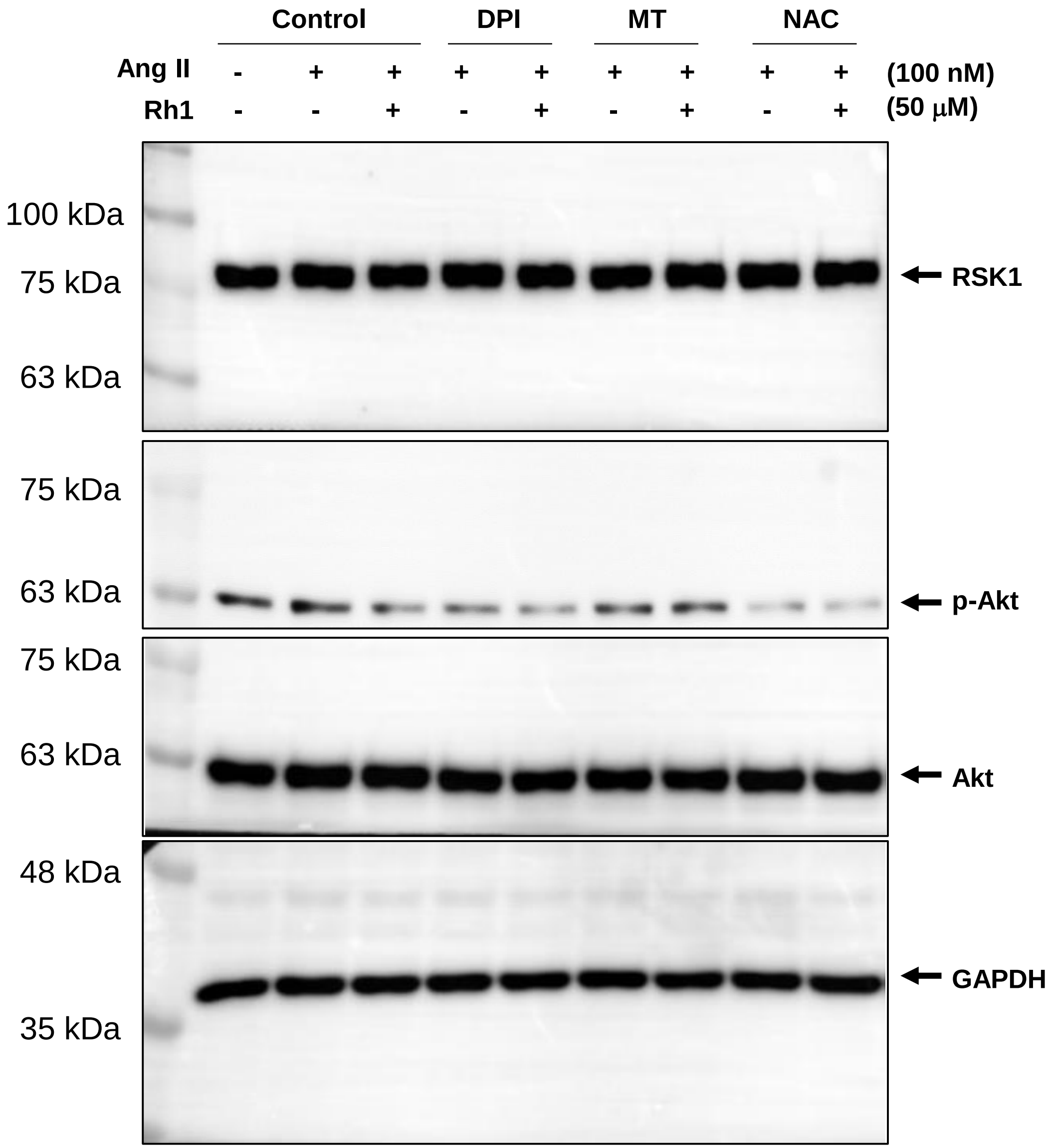

Control
DPI
MT
NAC
Ang II - + + + + + + + + (100 nM)
Rh1 - - + - + - + - + (50 μM)
100 kDa
75 kDa
63 kDa
RSK1
75 kDa
63 kDa
p-Akt
75 kDa
63 kDa
Akt
48 kDa
35 kDa
GAPDH

Figure 8E

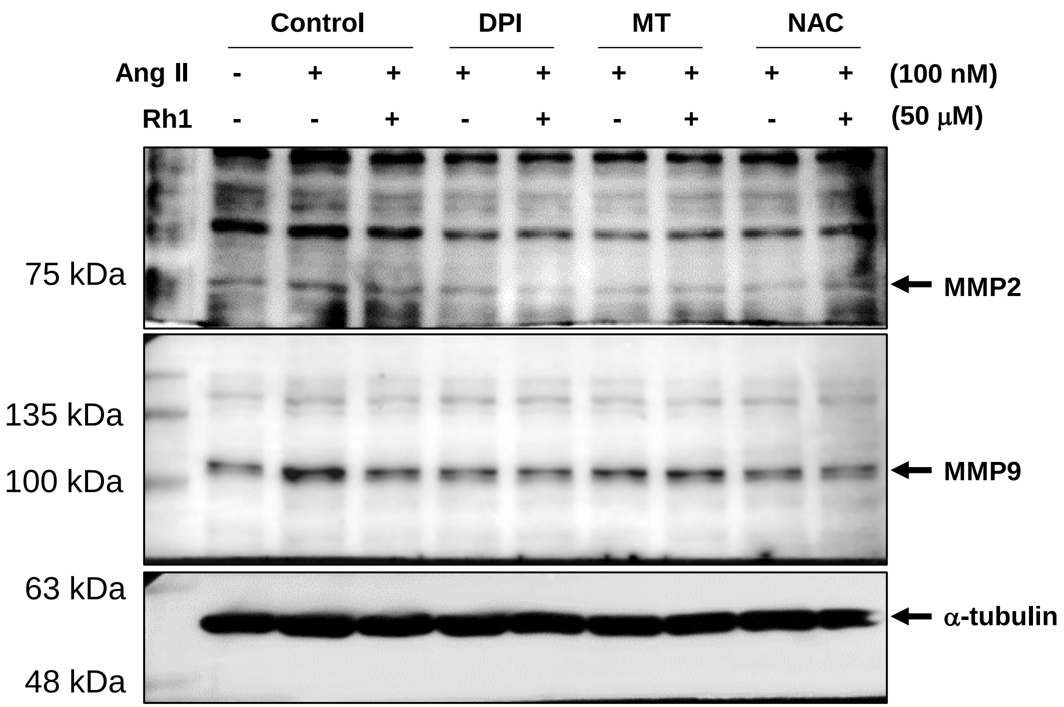

Control
DPI
MT
NAC
Ang II - + + + + + + + + (100 nM)
Rh1 - - + - + - + - + (50 μM)
75 kDa
MMP2
135 kDa
100 kDa
MMP9
63 kDa
48 kDa
α-tubulin

Figure 9A

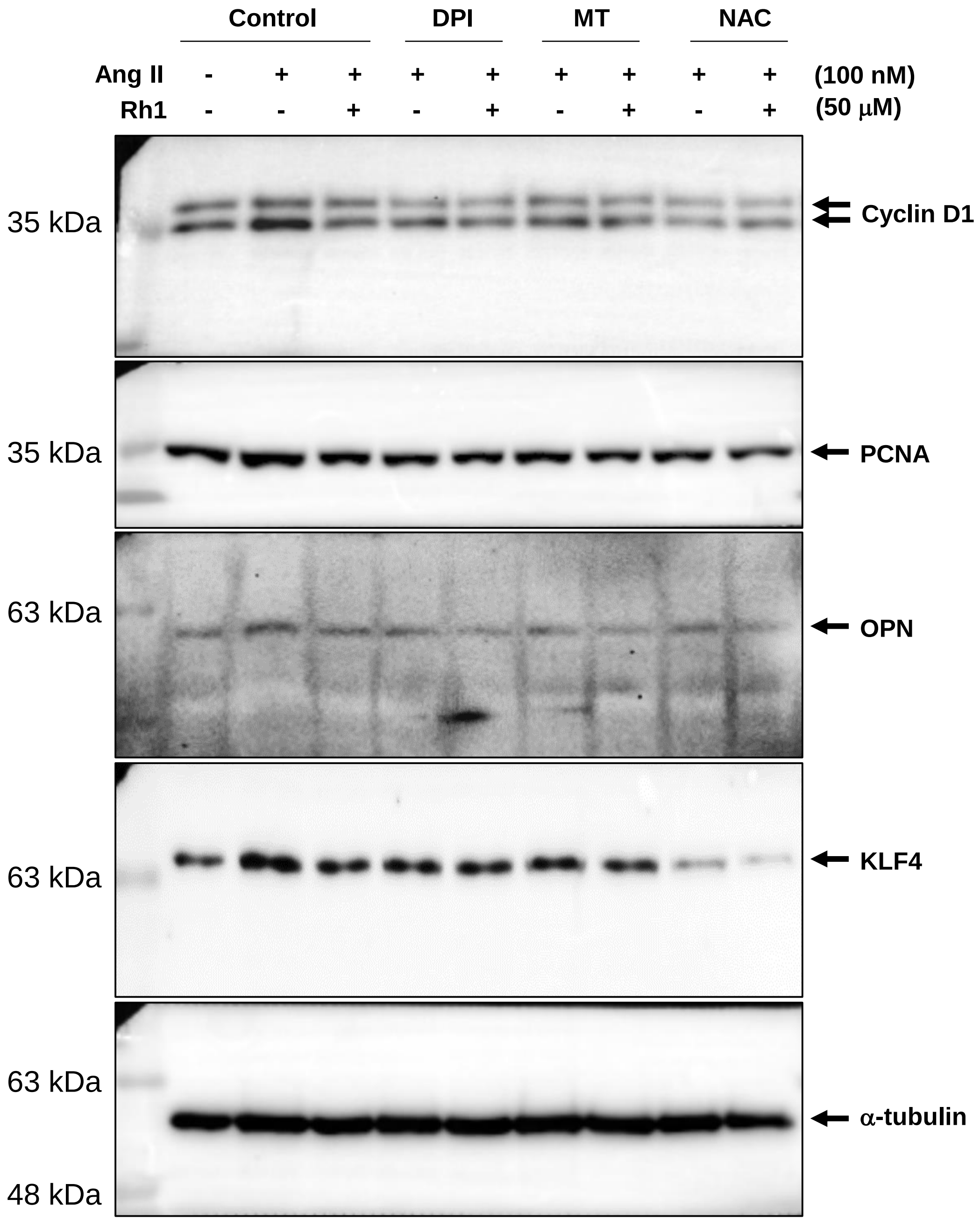

Figure 9F

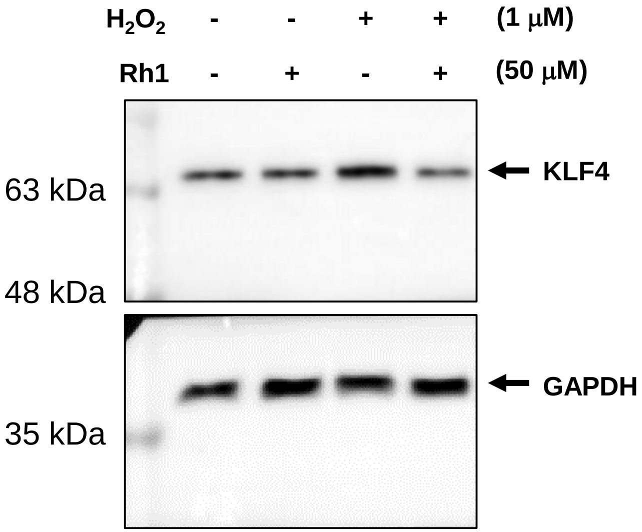

Supplement: Supplementary file 1 [file antioxidants-11-00643-s001.zip › File S1.pdf]
